# Recent Advances in Pyrazole-Based Cholinesterase Inhibitors: Medicinal Chemistry Perspectives from 2020 to 2025

**DOI:** 10.3390/ph19071079

**Published:** 2026-07-13

**Authors:** Lalsu Yeysin, Deniz Akın, Süleyman Çalışkan, Elvan Hasanoğlu Özkan, Hamada Hashem, Suleyman Akocak, Stefan Bräse, Servet Çete

**Affiliations:** 1 Institute of Science, Gazi University, Ankara 06500, Türkiye; lalsu.yeysin@gazi.edu.tr; 2 Department of Chemistry, Faculty of Science, Gazi University, Teknikokullar, Ankara 06500, Türkiyeehasanoglu@gazi.edu.tr (E.H.Ö.); 3 Department of Material Processing Technologies and Polymer Technology, Kırıkkale Vocational School, Kırıkkale University, Kırıkkale 71450, Türkiye; 4 Department of Pharmaceutical Chemistry, Faculty of Pharmacy, Sohag University, Sohag 82524, Egypt; 5 Department of Pharmaceutical Chemistry, Faculty of Pharmacy, Adıyaman University, Adıyaman 02040, Türkiye; 6 Institute of Biological and Chemical Systems Functional Molecular Systems (IBCS-FMS), Karlsruhe Institute of Technology (KIT), Kaiserstrasse 12, 76131 Karlsruhe, Germany

**Keywords:** pyrazole, acetylcholinesterase, butyrylcholinesterase, Alzheimer’s disease, drug design

## Abstract

Pyrazole derivatives have attracted considerable interest in medicinal chemistry as adaptable frameworks for developing cholinesterase inhibitors, owing to their advantageous physicochemical properties and structural flexibility. The heteroaromatic characteristics of the pyrazole core allow for various substitution patterns, promoting selective interactions with both the catalytically active site (CAS) and the peripheral anionic site (PAS) of cholinesterase enzymes. These attributes enable pyrazole-based drugs to be viable candidates for the therapy of cognitive disorders, especially Alzheimer’s disease. This study aims to systematically describe medicinal chemistry studies on pyrazole-based cholinesterase inhibitors conducted from 2020 to 2025. The focus is on structural alterations of the pyrazole core and their impact on the inhibitory action against acetylcholinesterase (AChE) and butyrylcholinesterase (BChE) using structure–activity relationship (SAR) analysis. Recent advancements in in vitro enzymatic inhibition studies, molecular docking, kinetic analysis, ADME predictions, and multi-target-directed ligand (MTDL) techniques are rigorously evaluated to elucidate trends in potency, selectivity, and drug-like characteristics based on information retrieved from three search engines: Scopus, PubMed, and Google Scholar. This review addresses significant challenges in pharmacokinetics, blood–brain barrier permeability, and safety while delineating prospects for integrating rational design, computational modeling, and biological validation to expedite the development of clinically relevant pyrazole-based cholinesterase inhibitors for Alzheimer’s disease.

## 1. Introduction

Neurodegenerative disorders are long-term diseases that are characterized by progressive damage to neuronal structures and their activities. Among them, the most widespread ones are Alzheimer’s disease (AD) and Parkinson’s disease [1]. AD is an important public health issue globally, whose symptomatology, which includes impairment of memory and cognitive functions and causes behavioral disruptions, immensely affects the performance of daily tasks [2]. Despite extensive research conducted over several decades, the complexity of the exact molecular pathways involved in AD persists [3]. The pathophysiology of the disease has been attributed to several biochemical pathways, including amyloid aggregation, tau hyperphosphorylation, oxidative stress, and neuroinflammatory processes [4]. The cholinergic hypothesis is one of the oldest, most familiar, and empirically supported theories explaining the cognitive impairments in AD [5]. According to this hypothesis, the decline in acetylcholine (ACh) results from the loss of cholinergic neurons and the continued presence of cholinesterase, which overhydrolyzes ACh [6]. The ensuing exponential decrease in the level of ACh triggers the cognitive and memory deficiencies, which qualify cholinesterase inhibitors as a crucial drug in the treatment of AD symptoms [7].

Acetylcholinesterase (AChE) and butyrylcholinesterase (BChE) are two enzymes that mediate cholinergic neurotransmission [8]. These hydrolases are crucial for completing neuronal signaling and maintaining cholinergic equilibrium [9,10]. AChE is a serine hydrolase, a specialized protein mostly located on the postsynaptic membranes of both peripheral and central cholinergic synapses [11]. It dissolves ACh in about a microsecond, thus facilitating a very complex modulation of synaptic transmission, temporal fidelity, and neural plasticity. BChE, on the other hand, has broader substrate specificity and is found in a variety of tissues, including the liver, plasma, and glial cells [12,13]. The physiological contribution of BChE to ACh regulation is small; nevertheless, when AChE activity decreases in advanced AD, BChE plays an increasingly important modulatory role.

Recent molecular studies have established marked differences in kinetic characteristics, substrate-binding structures, and inhibitor sensitivities between AChE and BChE, highlighting their pharmacological differences. This distinction is very important in diagnostic assessment as well as in the treatment of neurodegenerative disorders [14]. There is empirical evidence that AChE activity gradually decreases as AD progresses, whereas BChE activity increases [15]. These enzymatic disequilibria not only interfere with cholinergic signaling but also place BChE in charge of maintaining the basal synaptic ACh concentration in the later stages of the disease, making BChE a more relevant drug development target. The dual-enzyme dynamics of AChE/BChE are, therefore, central to the mechanistic concept of AD pathophysiology, and they were utilized in the design of next-generation, more potent cholinesterase inhibitors [16].

Multiple related pathways describe the therapeutic advantages of simultaneously inhibiting AChE and BChE. Initially, dual inhibition is more effective in preserving synaptic ACh reserves, leading to immediate improvements in memory performance and cognitive capacities [17]. Secondly, in the late AD stage, the decrease in AChE activity can be offset by BChE activity, which mitigates the steady decline in treatment efficacy associated with disease progression [18]. Thirdly, dual inhibitors can serve as a model in medicinal chemistry for improving blood–brain barrier permeability while minimizing peripheral side effects through a balanced distribution and enzyme-selectivity profile [19,20,21].

In the context of modern medicinal chemistry, dual AChE/BChE inhibitors combine the design principles of traditional small-molecule design with multi-target directed ligand (MTDL) strategies [22]. The main aim of structure–activity relationship (SAR) studies in this field is to balance both catalytic and peripheral binding-site interactions in the two target enzymes. Furthermore, there exists potential not only to reduce clinical symptoms but also to exert direct neuroprotective effects on the underlying pathogenic mechanisms via MTDL analogs that integrate cholinesterase inhibition with additional activities, including antioxidant properties, metal chelation, or amyloid-beta modulation [23]. Nevertheless, these multifunctional modalities face challenges related to improved pharmacokinetics and toxicology that must be considered [24]. The therapeutic efficacy of dual inhibitors in clinical contexts must extend beyond simple symptom treatment to encompass cognitive assessment outcomes, illness progression metrics, long-term safety profiles, and overall daily functional performance. There could be benefits to extensively describing the dynamics of changes in enzyme activity in preclinical and translational models, as this would allow the selection of patient subsets that would most likely respond to dual-inhibition targets, thereby integrating therapeutic studies into the paradigm of personalized medicine.

In summary, dual inhibition of AChE and BChE is a better approach than the traditional cholinergic hypothesis since it accounts for the enzymatic changes that occur during disease development and allows the benefits for cognitive changes to be sustained longer [24]. However, to support this strategy, the application of specialized pharmacological models, the implementation of carefully planned preclinical studies, and the clinical trial certified using the relevant biomarkers are required [25,26].

**Figure 1:**
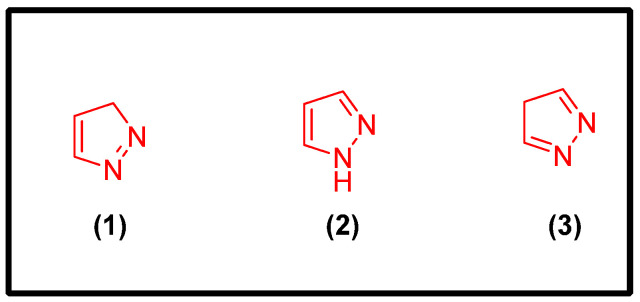
Pyrazole and its tautomer structure.

**Figure 2:**
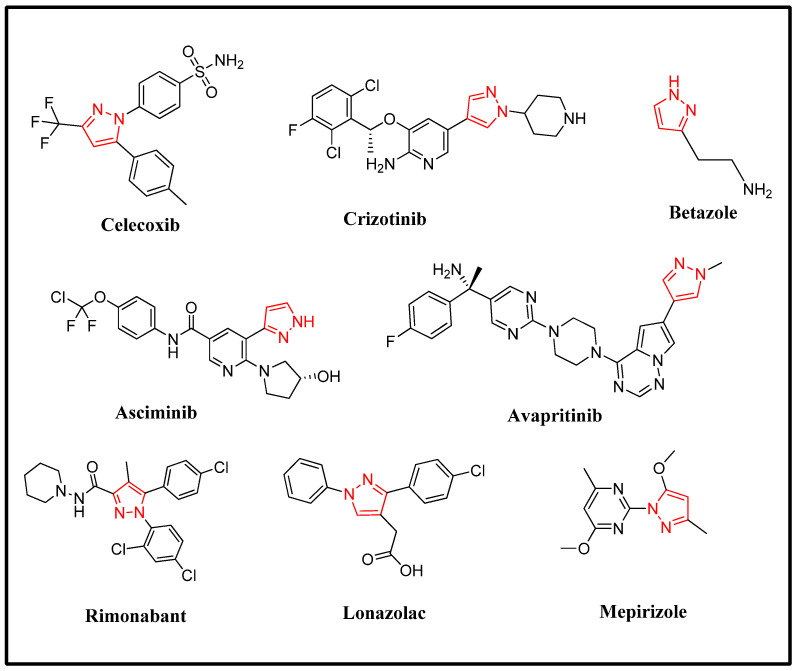
Some examples of pharmaceutical drugs containing the pyrazole moiety.

The pyrazole rings, owing to the presence of two neighboring nitrogen atoms, exist in three distinct tautomeric forms (Figure 1), which are poised to play a pivotal role in drug design due to their strong biological potency and potential therapeutic benefits (Figure 2). These heterocyclic five-membered rings have been converted into a range of bioactive products that exhibit antiviral, anticancer, antidepressant, anti-inflammatory, and antioxidant properties [27,28,29,30,31,32]. Their ability to engage with a wide range of molecular entities provides them with significant flexibility in regulating diverse biological functions. The introduction of a pyrazole moiety is often necessary to enhance the therapeutic potential of pharmacological agents. Pyrazoles have therefore become an integral part of medicinal chemistry, especially in the development of agents that can regulate the activity of enzymes/receptors [33]. The therapeutic value of pyrazole derivatives necessitates the systematic synthesis of novel derivatives with greater selectivity and efficacy; thus, this exercise is a research priority of high order [34]. The possibility of selectively modifying these compounds to inhibit cholinesterases can be used to treat disease related to cholinesterase dysfunction in pathological conditions [35]. Moreover, further exploratory studies of pyrazole-containing architectures are expected to yield next-generation therapeutics that are more selective and have fewer off-target effects [36].

The primary objective of this review is to carefully combine medicinal chemistry investigations on pyrazole-based cholinesterase inhibitors completed from 2020 to 2025. This review examines the structural modifications in the pyrazole core, their impact on AChE/BChE activity (SAR analysis), in vitro enzymatic inhibition, molecular docking studies, kinetic mechanisms, ADME predictions, MTDL strategies, and their therapeutic potential in Alzheimer’s disease, along with perspectives on clinical translation and future challenges. This review aims to identify current developments and outline a framework for future drug development initiatives in Alzheimer’s disease by describing recent advancements in pyrazole-based inhibitor research.

## 2. Structural Characteristics of Pyrazoles

**Figure 3:**
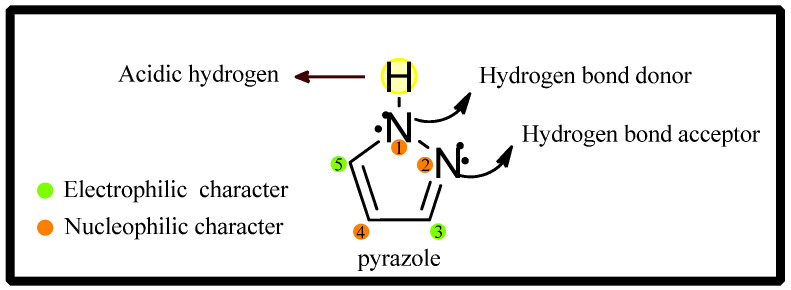
Structural and electronic features of pyrazole.

The presence of two neighboring nitrogen atoms in the five-membered pyrazole ring distinguishes it from other heterocyclic systems by providing an exceptional electronic structure. The adjacent nitrogen atoms create a substantial molecular dipole, exerting a pronounced directional influence on the molecule’s chemical and physical properties (Figure 3). Pyrazole possesses an almost aromatic structure, as the bond lengths are not perfectly equalized and the resonance structures exhibit varying degrees of stabilization. This represents an intermediate degree of aromaticity, balancing chemical stability and reactivity, and is a defining trait of the pyrazole framework. The acidity/basicity of pyrazole is directly dependent upon the electronic configuration of the molecule. Pyrazole, with an estimated pKa of approximately 2.5, is a comparatively weaker base than imidazole; yet, it remains highly susceptible to protonation by strong inorganic acids. Tautomerism arises from proton transfer between the two nitrogen atoms; the tautomeric equilibrium is affected by solvent polarity and the electronic characteristics of substituents. Optimizing tautomer populations using Hammett parameters is essential, particularly in medicinal chemistry, where tautomeric preferences influence chemical recognition and binding. Synthetic transformations also rely on tautomerism, and regioselective alkylation happens on either nitrogen atom under the influence of solvent and substituent effects [37,38,39].

Pyrazole is a remarkable heterocycle owing to its aromaticity and ability to generate hydrogen bonds, rendering it highly compatible with biological systems. It can establish π-π stacking interactions with aromatic amino acid residues, namely phenylalanine, tyrosine, and tryptophan. The dual hydrogen-bond donor-acceptor characteristics facilitate precise identification inside enzyme active sites, hence enhancing binding specificity. Pyrazole should be regarded as more than just an aromatic ring in contemporary medicinal chemistry, catalysis, and molecular recognition; it should be viewed as a multifunctional platform that integrates proton mobility, resonance stabilization, hydrogen-bonding motifs, and pharmacophoric versatility within a single skeletal framework [37,39,40,41].

### 2.1. Importance in Medicinal Chemistry

The pyrazole ring is crucial in drug research because it can alter its shape and electronic characteristics. The unequal distribution of electrons in nitrogen atoms forms a scaffold that can both accept and lose electrons, enabling dynamic interactions within enzyme microenvironments. This bidirectional acid-base action allows pyrazole-based pharmacophores to adopt a multiplicity of complementary interaction mechanisms; hence, their bioactivity is so extensive. Pyrazole not only suits a single type of target but also has a modular structure that can be modified to accommodate various electronic and topological purposes [34]. This flexibility provides bioisosteric features, allowing controlled variation in electrical density and polarity without altering the ring’s size or shape. This leads to enhanced pharmacokinetic characteristics [33]. The conjugated aromatic structure of pyrazole is metabolically rigid and, therefore, naturally resistant to oxidative degradation. The scaffold exhibits metabolic stability by preventing potential oxidation of cytochrome P450 due to resonance stabilization of the N-N bond. Substitution with electron-withdrawing groups or spatially occupying entities is strategic for preserving the site prone to subsequent oxidation, thus enhancing its ability to survive in the body. Consequently, pyrazole provides controlled polarity, lipophilicity, and metabolic stability for lead development [42].

The enzyme-binding properties of pyrazole are significant because they can interact with multiple pathways sequentially. π-π stacking interactions with aromatic residues frequently serve as the primary anchoring interaction, subsequently reinforced by directed hydrogen bonding. With two heteroatoms present, ligands can establish extended interactions rather than a single binding contact. The ligand may become increasingly rigid due to intramolecular hydrogen bonding, thereby reducing the loss of conformational entropy during binding and enhancing the ligand’s optimal fit within enzyme cavities. A molecular modeling study indicates that pyrazole analogs can modify the charge distribution in an enzyme’s active site, particularly at the aromatic and negatively charged surface motifs. This is useful for understanding electrostatic resonance [36,37,38]. Three principal chemical attributes contribute to the pharmaceutical efficacy of the pyrazole core: (i) its compact, modular aromatic structure facilitates precise electronic modulation without modifying bioisosteric geometry; (ii) its resonance-stabilized, conjugated framework confers resistance to oxidative metabolism; and (iii) its resonance-stabilized, conjugated architecture allows for π-π interactions and hydrogen bonding between its dual nitrogen sites, thereby stabilizing binding geometry. These attributes enable pyrazole to be a versatile pharmacophore that combines binding stability, metabolic stability, and target selectivity within a single chemical framework.

### 2.2. Common Synthetic Approaches

#### 2.2.1. Condensation of Hydrazines with 1,3-Diketones or β-Dicarbonyl Compounds

The condensation of hydrazine derivatives with 1,3-diketones and 2-carbonyl compounds is a highly reliable and reproducible method for synthesizing the pyrazole ring. This classical Knorr-type condensation has been reconsidered in recent years through the utilization of alternative catalytic surfaces, novel experimental systems, and micro-environmental technologies aimed at reducing energy input while enhancing the reaction rate and selectivity. The following articles summarize research that has improved pyrazole production across various reaction conditions.

**Figure 4:**
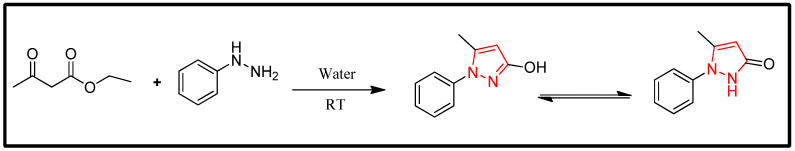
Synthetic method used to produce pyrazoles by Jana et al. [43].

The latest advancement in condensing systems, which diverges from conventional methods, is the ultrafast condensation system developed by Jana and co-workers, which utilizes aqueous microdroplets [43]. Reactions combining traditional 1,3-diketones such as ethyl acetoacetate, 1,3-diphenyl-1,3-propanedione, or heptane-3,5-dione with hydrazine hydrate or phenylhydrazine required minutes to hours in the bulk phase [44,45,46], such as the use of acetic acid as a solvent and refluxing for 24–36 h at around 122 °C. Conditions like this might affect the whole sustainability and energy efficiency of the process (Table 1), whereas in microdroplets, the reaction occurred within milliseconds (Figure 4). This remarkable acceleration was attributed to proton-rich interfacial environments, intense electric field strengths, and boundary-layer dynamics at the droplet surface. The high-yield pyrazole compounds were generated via rapid surface-confined nucleophilic addition and cyclization, consistent with the traditional Knorr mechanism, without the use of any external catalyst. NMR and mass spectrometry analyses unequivocally demonstrated that the chemical intermediates produced at the microdroplet interface were promptly converted into the five-membered pyrazole ring [43].

The study by Abdel Hafiz and colleagues provides a comparable example, illustrating the formation of pyrazole via the generation of 1,3-diketone intermediates from complex precursors, followed by cyclization with hydrazine [47]. This approach involves the solvent-free, microwave-assisted transformation of ethyl benzoylacetate and m-cresol into a 1,3-diketone via phenolic substitution. The next reaction of this diketone with hydrazine hydrate produced the corresponding pyrazole in substantial yield. Experimental programs have proposed a two-step synthesis strategy involving an initial hydroxyarylation of the diketone core, followed by a hydrazine-induced ring closure. The pyrazole derivatives were structurally validated using IR spectroscopy, which exhibited characteristic NH and aromatic C=N vibrations, and NMR spectroscopy, in which the pyrazolic proton appeared as a distinct singlet at 6–7 ppm, indicating successful cyclization [47] (Table 1).

**Figure 5:**
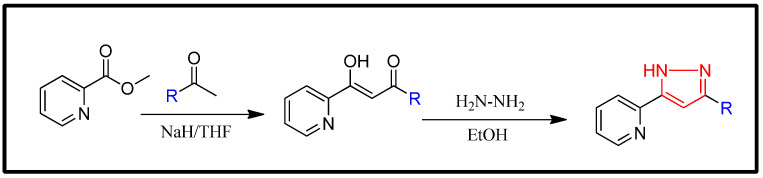
Synthetic scheme for pyridine–pyrazolate derivatives [48].

Javaid and colleagues created a methodology that centers around pyrazole condensation within functional ligand chemistry [48]. This approach involved the synthesis of picolinate-derived 1,3-diketones, which were then reacted with hydrazine hydrate to produce pyridine–pyrazolate frameworks (Figure 5). Due to the coordination capability of the pyrazolic nitrogen, these structures were subsequently utilized in the synthesis of boron complexes. The experimental methodology entailed refluxing the condensation mixture in ethanol, typically using a two- to threefold excess of hydrazine. Significantly, the majority of products were acquired in substantial yields without the necessity for column chromatography. These findings emphasize the hydrazine–diketone condensation as a fundamental reaction for heterocycle synthesis and a versatile foundation for advanced applications in coordination and materials science [48].

#### 2.2.2. Multicomponent and Microwave-Assisted Syntheses

**Figure 6:**
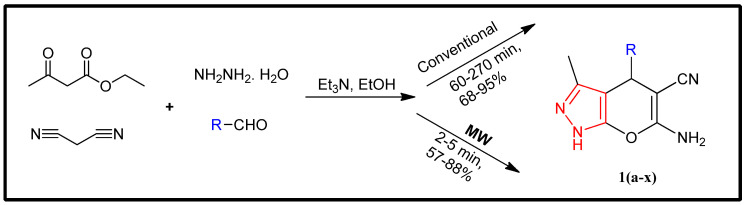
Traditional heating and microwave-assisted synthesis of pyrano [2,3-*c*]pyrazoles [50].

In recent years, methodologies that combine multicomponent reactions with microwave-assisted activation have gained considerable significance in the synthesis of heterocyclic frameworks featuring pyrazole and its derivatives. A significant advantage of these approaches is the substantial decrease in reaction times; transformations that typically necessitate minutes to hours with conventional heating can be accomplished within minutes or even seconds under microwave irradiation, frequently yielding high results. Ahmad and colleagues’ microwave-assisted syntheses of pyrano [2,3-c]pyrazoles, concentrating on three- and four-component systems, exemplify this recent progress [49]. Aldehydes, malononitrile, β-dicarbonyl compounds, and hydrazine derivatives were transformed into the desired products in a single step by 5–6 min of microwave heating (Figure 6). This method clearly illustrates kinetic acceleration and reduced energy consumption. The remarkable isolated yields of 68–95% demonstrate that microwave irradiation not only reduces reaction times but also enables the rapid production of essential intermediates in multicomponent condensation processes, resulting in a more effective closure of reaction pathways. NMR and IR spectroscopic structural characterization yielded diagnostic signals and bands indicative of pyrazole ring formation, thereby validating the efficacy of this expedited synthetic approach [50].

**Figure 7:**
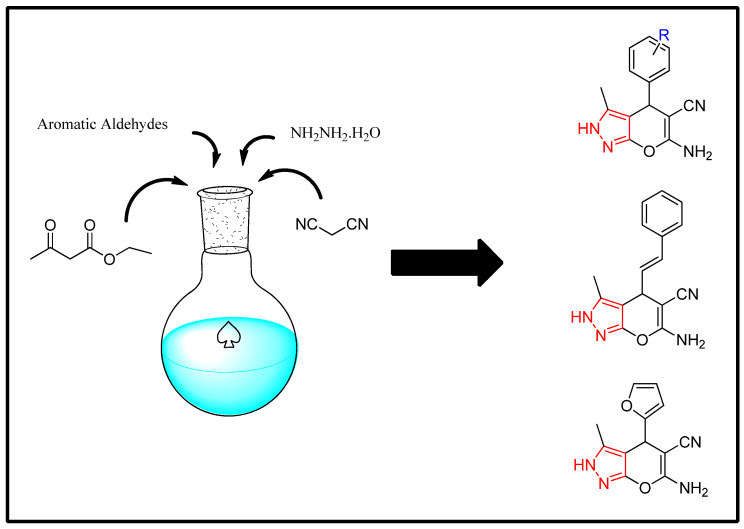
Microwave-assisted four-component synthesis of pyranopyrazoles [51].

The microwave-assisted four-component synthesis of pyranopyrazoles, developed by Yallappa and colleagues, further corroborates the efficacy of this reaction principle across many structural systems [51]. The reaction concluded in roughly 5 min and consistently produced high yields of 85–90% across nearly all tested conditions (Figure 7). These data provide compelling evidence that microwave irradiation significantly accelerates the rate of multicomponent processes. The lack of observable intermediates under standard thermal circumstances, in contrast to their rapid conversion to products under microwave irradiation, indicates that dielectric heating creates localized, high-energy microenvironments that expedite ring-closure reactions. Yallappa’s investigation showed that reactions in polar protic solvents, especially methanol, proceeded most quickly and effectively, indicating that dipole moment alignment under microwave fields significantly affects reaction kinetics [51] (Table 2).

**Figure 8:**
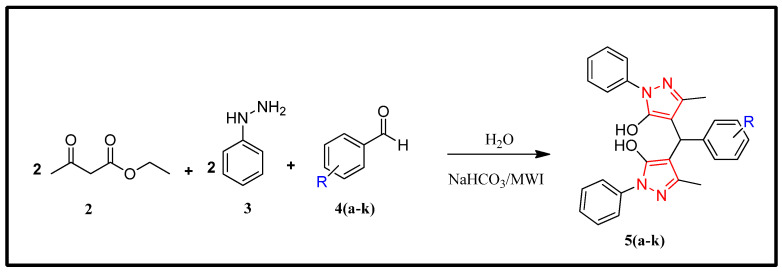
Microwave-assisted synthesis of bis-pyrazole analogs **5**(**a**–**k**) [55].

The bis-pyrazole synthesis reported by Kumar and co-workers represents another noteworthy application of microwave technology, combining multicomponent strategies with green chemistry principles [55]. In this approach, benzaldehyde, ethyl acetoacetate, and phenylhydrazine were transformed into high-purity bis-pyrazole products in aqueous media using only 20 min of microwave irradiation (Figure 8). The excellent yields obtained (93–97%) highlight the industrial potential of this methodology to produce compounds **5**(**a**–**k**). Activation of aromatic aldehydes under microwave conditions was shown to be particularly effective for the rapid construction of pyrazole-based molecular libraries. Moreover, conducting the reaction in water not only reduces the environmental impact but also creates proton-rich microenvironments that facilitate hydrazone formation and favor subsequent cyclization. As a result, the sequential ring-closure steps leading to bis-pyrazole frameworks proceed with high selectivity and purity [55].

#### 2.2.3. Recent Green Chemistry Strategies

Current developments in the production of pyrazoles indicate a single step towards greener methods that conform to the guidelines of green chemistry. Recent practices emphasize reduced solvent use, lower energy consumption, elimination of hazardous catalysts, and increased atom economy, demonstrating that sustainable synthesis is achievable without compromising reaction efficacy.

**Figure 9:**
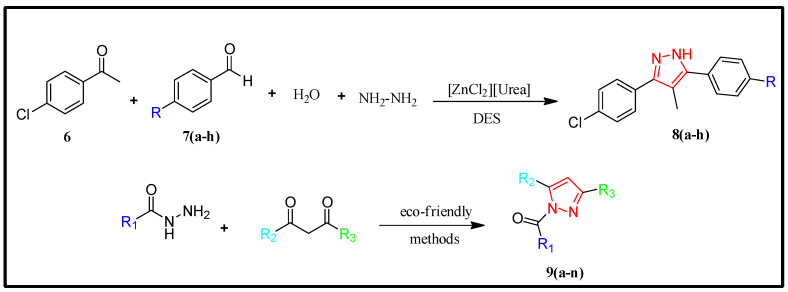
Synthesis of pyrazole analogs **8**(**a**–**h**) and **9**(**a**–**n**) under green conditions [56,57].

A good example is the multicomponent method, realized using a deep eutectic solvent (DES) by Mohamed and Abdullah, in which a urea/ZnCl_2_ DES was used to hasten the synthesis of pyrazole under mild microwave conditions (Figure 9). The good hydrogen-bonded complex formed by the DES promoted successful carbonyl activation, and thus the reaction took place at a low temperature with minimal waste. This methodology focuses on the effectiveness of solvent-free, energy-saving systems for making heterocycles [56].

The study by Świetczak et al. provided additional information by comparing ultrasonic irradiation, mechanochemical ball-milling, and microwave activation for synthesizing pyrazole (Figure 9). Their results revealed that solvent-free ball-milling is exceptionally efficient for sterically challenging substrates and that mechanochemistry is becoming increasingly important as a sustainable alternative. The discovery of aggregation-induced emission effects in specific derivatives demonstrates the utility of the chemicals produced under environmentally friendly conditions [57].

Merzouki and co-authors developed a catalyst-free method for aminating (3,5-dimethylpyrazol-1-yl)methanol with various amines at low temperatures. This method generated mono- and bis-pyrazole products in moderate to high yields, ensuring a small environmental footprint and convenient accessibility. Taken together, these discoveries have shown that modern pyrazole production has advanced towards multi-purpose, eco-friendly procedures, in which classical condensation reactions, energy-driven methods, and catalyst-free processes are used synergistically. This unification makes the pyrazole scaffold an exemplary system for green heterocyclic synthesis and shows that convergent green methods will likely continue to expand in research [58].

## 3. Mechanistic Basis of Cholinesterase Inhibition

**Figure 10:**
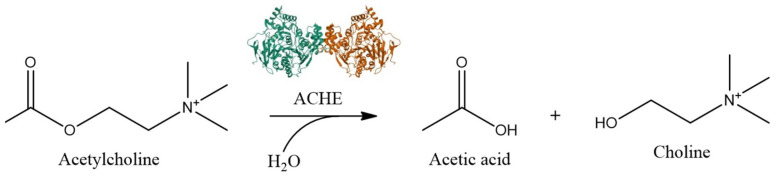
Hydrolysis of acetylcholine by acetylcholinesterase (AChE).

There are two main types of the cholinesterase enzyme family: AChE and BChE. These enzymes exhibit substantial differences in their biological activities despite their striking structural similarities. The main role of AChE is to rapidly hydrolyze acetylcholine at nerve–muscle synapses in the central nervous system, playing a regulatory role at the end of a neurotransmission (Figure 10). AChE is often referred to as the true cholinesterase because of its very high catalytic potency. It is found in muscular, neuronal, and hematopoietic tissues and is required to relay proper physiological signals [59].

BChE is a homologous enzyme that is mainly present in blood plasma and was previously known as “pseudocholinesterase”. Compared to AChE, BChE is also more substrate-specific, more tolerant to mutational changes, and is mostly generated in the liver. Although its physiological role was long unidentified, molecular modeling and simulation studies recently suggest that BChE may have diverged from AChE and is a general purifying enzyme, thus playing an indirect but constant role in neurotransmission. This theory is supported by structural congruence in the active sites of the two enzymes, similar substrate and ligand-binding affinities, and an approximate 65 percent sequence homology [59].

AChE and BChE belong to the serine hydrolase family, which catalyzes the hydrolysis of esters through a conserved catalytic triad of residues, Ser-His-Glu. This triad in human AChE consists of Ser203, His447, and Glu334, which act in synergy in the hydrolysis reaction. Hybrid QM/MM studies have elucidated the complex chemical process, showing that the first reaction involves a nucleophilic attack on the Oγ atom of Ser203 on the carbonyl carbon of acetylcholine. During this process, His447 acts as an inert base, accepting a proton from Ser203 and hydrogen bonding with the protonated histidine via Glu334, thus lowering the activation energy barrier [60].

AChE possesses a catalytic triad situated at the base of a deep, narrow, almost aromatic cavity measuring approximately 20 Å. The α/β-hydrolase core of the enzyme, as elucidated by Hung and colleagues, shows that the arrangement of 12 β-strands and 14 α-helices produces a significant dipole moment, facilitating the orientation of substrates towards the active region [61]. The aromatic residues within the gorge, such as Trp86, Tyr337, Phe330, and Tyr121, participate in cation-pi interactions with the positively charged choline moiety, creating a tunnel that channels substrates to the catalytic core. Kinetic studies suggest that the Phe330-Tyr121 pair, situated in the center of the gorge, may temporarily close to function as a bottleneck, regulating the influx of substrates into the channel [62]. Computational research conducted by Xu and colleagues has demonstrated that this aromatic channel has an active role in facilitating access by creating a favorable electrostatic field rather than serving just as an inactive channel [63].

Although BChE and *E. coli* use the same catalytic mechanism, BChE has a distinct binding architecture, characterized by a broader gorge and lower density of aromatic residues. According to Gambardella et al., BChE contains a significantly larger active-site volume than AChE. The change in Phe295 and Phe297 in AChE to valine and leucine residues in BChE mainly explains this difference [64]. This changes the structure to give greater tolerance to larger ligands, and the idea that BChE is a more universal purifying enzyme is confirmed. The PAS adjacent to the entrance of the AChE gorge acts as the main binding site for substrates and inhibitors, playing an important role in determining the enzyme’s kinetics [64].

Cheung and colleagues have demonstrated the transient binding of substrates to the PAS for up to 10^−9^–10^−8^ s, which is sufficient as a residence period to ensure proper orientation before catalysis, thereby enhancing hydrolytic efficiency [65]. The PAS would also act as a selective binding site for some inhibitors and regulate substrate traffic. As an example, thioflavin T acts as an inhibitor by blocking the entrance to the gorge, whereas a chemical such as dihydrotanshinone I has a high affinity for binding to the PAS. The locus of hydrolytic chemical processes is the CAS, located at the bottom of the gorge. QM/MM studies have explained the nucleophilic attack of Ser203, proton transfer via His447, and stabilization by Glu334 at atomic resolution. The oxyanion hole within the CAS stabilizes the negatively charged transition state, thereby reducing the activation energy barrier and enabling AChE to hydrolyze tens of thousands of substrate molecules per second. Quantitative studies show that this stabilization lowers the energy of the transition state by approximately 6–8 kcal/mol and provides a structural basis for the high level of catalysis exhibited by the enzyme. The coordinated action of the PAS and CAS is the key to AChE activity [59,62]. Kinetic studies also support the idea that inhibitors that interact with both PAS and CAS often exhibit mixed or non-competitive inhibition, reflecting interactions with free enzyme and enzyme–substrate complexes [18]. The PAS controls the acquisition and alignment of substrates, and the CAS controls the chemical transformation. After comparing Hung and Xu’s dipole moment studies, one will notice that the outstanding performance of AChE is due not only to catalytic triad chemistry but also to a coordinated, enzyme-wide electrostatic and structural process [61,63].

### 3.1. Binding Modes of Pyrazole-Based Inhibitors

AChE includes a small, approximately 20 Å deep aromatic groove with numerous aromatic amino acid residues. Pi-pi stacking, hydrogen bonding, and hydrophobic interactions are particularly observed in this area. Pyrazole-containing molecules are especially effective in the active involvement of all three types of interactions in this gorge because of their planar aromatic properties and ability to serve both as hydrogen-bond donors and acceptors. Persistent hydrogen-bond networks and hydrophobic anchoring, which are observed during molecular dynamics trajectories, have been correlated with stabilization of the ligand orientation and are generally viewed as indicative of long-residence binding modes [23].

#### 3.1.1. π-π Stacking with Trp84 and Phe330

AChE has aromatic residues that define its catalytic gorge, specifically Trp84 and Phe330, that play a significant role in stabilizing the ligand through π-π interactions. According to Shrestha and colleagues, phytochemical ligands form strong pi-pi stacking interactions, especially with Trp84 and Phe330, which play a major role in positioning the ligands at the optimal site and reducing the overall binding energy [66]. The results show that the best complementary qualities of the aromatic surfaces found in the gorge are found in inhibitors with conjugated aromatic systems, such as pyrazole compounds [66]. Sungthong et al. also found that Phe330, which is located at the canyon entrance, and Trp84, which is located in the catalytic pocket, stabilize the ligands through π interactions. They also emphasized that planar aromatic compounds exhibit a very high affinity to these residues [67]. The Trp84-Phe330 axis, as an essential aromatic interaction pathway between the PAS and the CAS, was highlighted as a key example for inhibitor design by Peitzika and Pontiki, who reviewed docking studies published in 2018–2022 [68]. Additional evidence comes from the work of Çetin et al., who reported substantial π interactions between pyrazole analogs and residues such as Trp286, Tyr341, and Phe295. Even though Trp84 and Phe330 were not directly implicated, the results further support the strong compatibility of the pyrazole derivatives with the aromatic structure of the AChE gorge [69].

#### 3.1.2. Hydrogen Bonding with Ser203 and His447

Messaad and colleagues discovered that compounds with a pyrazole ring are directly hydrogen-bonded to Ser203 and are stabilized in the vicinity of His447. Such interactions were known to be important in determining inhibitory potency [70]. In the same line, Abdelwahab et al. showed that the pyrazole-furan hybrids formed a hydrogen bond with Ser203, His447, and Glu334, thereby increasing the binding of the ligand with the catalytic triad [71]. Jaman et al. also emphasized the importance of His447 by examining epoxide derivatives capable of forming covalent bonds with this residue. Given the central role of His447 in proton transfer processes, the ligands that bind this residue may form both hydrogen and covalent bonds. Although pyrazole derivatives typically fail to form covalent interactions, their interactions with the electron-rich and cationic sites adjacent to His447 are mechanistically consistent with this notion [72]. Moreover, Son et al. showed that hydrogen bonds along the Ser203-His447 axis are significant for stabilizing the inhibitors in the catalytic site and maintaining consistent binding orientations [73].

#### 3.1.3. Hydrophobic Interactions with Tyr121, Phe290, and Tyr334

The hydrophobic core, comprising residues Tyr121, Phe290, and Tyr334 in the central part of the AChE gorge, holds the pyrazole derivatives in place. Shrestha et al. reported an intense hydrophobic interaction with these residues and emphasized that this threefold is a stabilizing hydrophobic core that extends the residence time of the ligands [66]. Merzoug et al. also found that AChE inhibitors interact with aromatic residues, namely, Tyr334 and Tyr341, in the hydrophobic plateau between the PAS and CAS. That area is of great importance for the ligand’s site of attachment [74]. Past similar important multiple-copy simulations using Van Belle et al. have shown that hydrophobic anchoring of phenylalanine and tyrosine residues controls the kinetics of ligand transport in the gorge [75]. Structural studies by Simeon et al. defined the hydrophobic sub-pockets of the active site of AChE, with the key hydrophobic stabilizers of the active site being Tyr121, Phe290, and Tyr334 of the active site, which are essential in inhibitor binding [76].

### 3.2. Literature Perspective on Docking and Molecular Dynamics

In recent years, the computational design of pyrazole-based AChE and BChE inhibitors has shifted from static (often termed) docking protocols to combined protocols, involving docking and molecular dynamics (MD) simulations. The docking experiment has since become a screening phase, and the temporal stability and dynamics of the enzyme–ligand complex are verified by running MD simulations. It has proven to be very effective with derivatives of pyrazole, pyrazolone, and pyrazole-1,3,5-triazine, which possess electron-rich structures and flexible binding modes.

**Figure 11:**
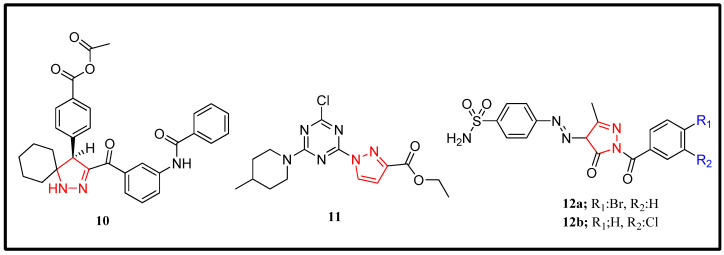
Active compounds from the literature [77,78,79].

El Alaouy et al. studied 3,5-diaryl-1H-pyrazolines and 3,5-spiropyrazolines and identified the most promising, which was named compound **10** (Figure 11) [77]. There were hydrogen-bonding interactions with Tyr124, Tyr72, and Ser293, indicating stability throughout the 100 ns molecular dynamics simulations. The low values in RMSD (1.5–2.0 Å) and the constant profile of the radius of gyration were indicators of a very coherent active site complex. The reported binding free energy (−151.225 kJ/mol) supported the ability of the optimized pyrazole cores to form deep stable coordination within the AChE active site [77]. A dynamic model proposed by Xue et al. is effective in inhibiting BChE using pyrazole-1,3,5-triazine derivatives [78]. The CAS/PAS most specific inhibitor, compound **11**, simultaneously reacted with both inhibitors (Figure 11). Regular contacts with Trp82 and His438 during molecular dynamics simulations, along with regular RMSD profiles, explained the observed preference of triazine-4-pyrazole hybrids for BChE [78]. The final study on sulfonamide-derived pyrazolones by Lolak et al. reports a strong correlation between static docking and modern dynamic binding methods. The 7.45–16.04 nM and 34.78–135.70 nM values of AChE and BChE, respectively, are the inhibitory values, which show the high compatibility of the core of pyrazole with the aromatic gorge (active compounds **12a** and **12b**) (Figure 11). Docking data illustrate π-π and hydrophobic interactions with Trp86, Phe338, and Tyr337, clearly replicating the traditional interactions commonly observed in pyrazole derivatives. This clarifies the strong affinity of the pyrazolone scaffold, based on its molecular aspect, which places it close to the aromatic triangle (Trp86, Phe337, Tyr337) [79].

## 4. Recent Advances in Pyrazole-Based Cholinesterase Inhibitors

Alzheimer’s disease is one of the focal points of current drug development efforts due to its progressive neurodegenerative nature and multifactorial pathogenesis. Within the cholinergic hypothesis, the inhibition of AChE and BChE remains a fundamental approach in symptomatic treatment [80,81,82,83,84,85]. In this context, small molecules containing heterocyclic cores are being intensively investigated due to their potential to adapt to the enzyme active site and establish multiple interactions. The pyrazole ring stands out as a privileged pharmacophore in the design of cholinesterase inhibitors due to its planar structure, π-π stacking ability, and hydrogen bond acceptor/donor properties.

Recent studies have revealed that pyrazole derivatives exhibit significant activity against AChE/BChE not only as standalone inhibitors, but also as a part of hybrid systems. This trend has paved the way for the repositioning of pyrazole-based molecules using the MTDL approach.

Despite the development of numerous synthetic strategies over the past decade, there is no comprehensive overview of the synthesis and effects of recent advances in the treatment of neurodegenerative diseases. This review first presents developments in the synthesis and functionalization of the pyrazole skeleton that have been published over the last decade (2020–2025). We then focus on the applications of these strategies in the development of therapeutics for neurodegenerative diseases, particularly Alzheimer’s disease and Parkinson’s disease (PD) [86].

### 4.1. Mono-Pyrazole Derivatives as Cholinesterase Inhibitors

As noted in the review by Kumar et al. [86], AChE and BChE inhibitors developed based on the cholinergic hypothesis for the treatment of Alzheimer’s disease constitute one of the most important research areas for pyrazole derivatives. The fact that a significant portion of pyrazole-based inhibitors reported in the literature exhibit Kᵢ or IC_50_ values in the nanomolar level demonstrates that this heterocyclic core has strong inhibitory potential against cholinesterase enzymes (Table 3).

**Figure 12:**
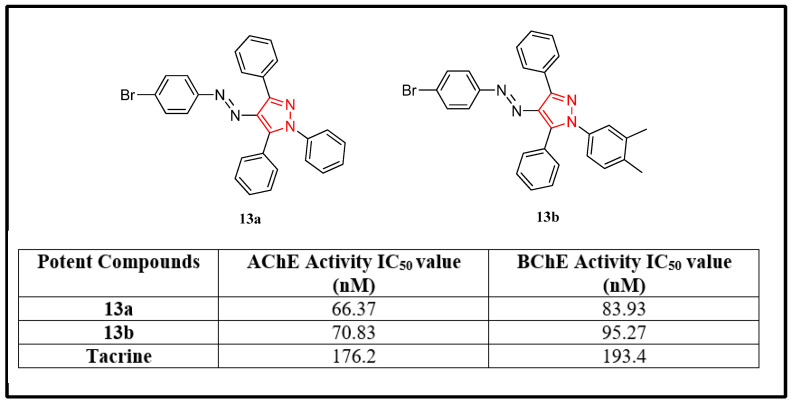
Pyrazol-4-yl-diazene derivatives (compounds **13a** and **13b**) as potent AChE and BChE inhibitors [87].

Turkan et al. (2018) reported that the pyrazol-4-yl-diazen derivatives they synthesized exhibited Kᵢ values from 44.66  ±  10.06 to 78.34  ±  17.83 nM for AChE, and 50.36  ±  13.88 to 88.36  ±  20.03 nM for BChE enzymes [87]. In this study, compounds **13a** and **13b** exhibited the highest inhibitory potency against AChE and BChE compared with the standard drug Tacrine (Figure 12). The inhibitory activity of these compounds was stated to be comparable to that of tacrine, which has previously been used clinically. However, the relatively low AChE/BuChE selectivity ratios of these compounds and their activity against α-glucosidase and carbonic anhydrase enzymes were considered potential limiting factors for clinical use [87].

**Figure 13:**
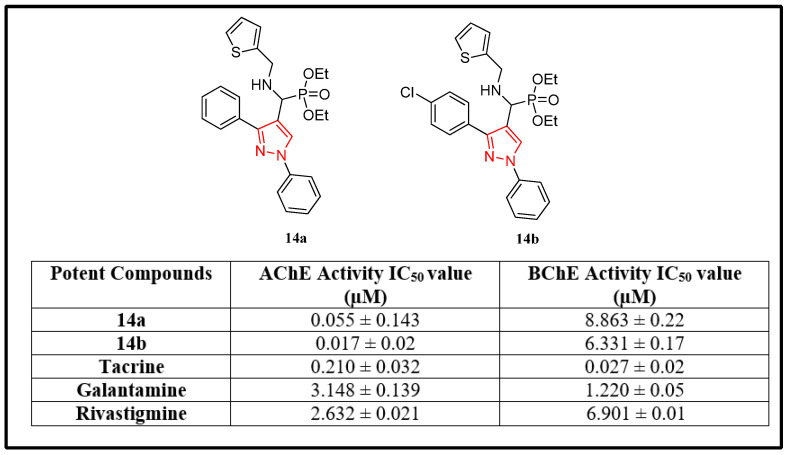
N-substituted pyrazole derivatives of α-aminophosphonates (compounds **14a** and **14b**) as potent AChE and BChE inhibitors [88].

Shaikh et al. reported that N-substituted pyrazole derivatives of α-aminophosphonates developed by them exhibited IC_50_ values in the range of 0.017–0.055 µM on the AChE enzyme. In contrast, they showed only a weaker inhibitory effect against BuChE at the micromolar level (Figure 13). More specifically, compounds **14a** and **14b** showed great inhibitory properties against AChE with IC_50_ values of 0.055 ± 0.143 and 0.017 ± 0.02 µM, respectively. Also, compounds **14a** and **14b** demonstrated high binding affinity to AChE (PDB: 1EVE; docking scores: −12.191 and −12.158 kcal·mol^−1^, respectively), consistent with their inhibitory potencies. According to the molecular docking results, it was shown that compound **14a** associated with Asp72 and Ser122 via hydrogen bonding and with Tyr121 through π-π stacking contact at the PAS. Interaction with Phe330 via π-π stacking at the anionic subsite and with Phe333 at the acyl binding pocket through π-π stacking was also noted. Compound **14b** similarly engaged at the PAS with Asp72, Tyr121, and Ser122 via hydrogen bonding, while interacting with Tyr334 through π-π stacking. Compound **14b** linked with the acyl binding pocket via π-π stacking with Phe330 and connected with the anionic subsite through π-π stacking with Phe331. These results suggest that these compounds may offer greater AChE selectivity than commercially available drugs such as galantamine and rivastigmine [88].

**Figure 14:**
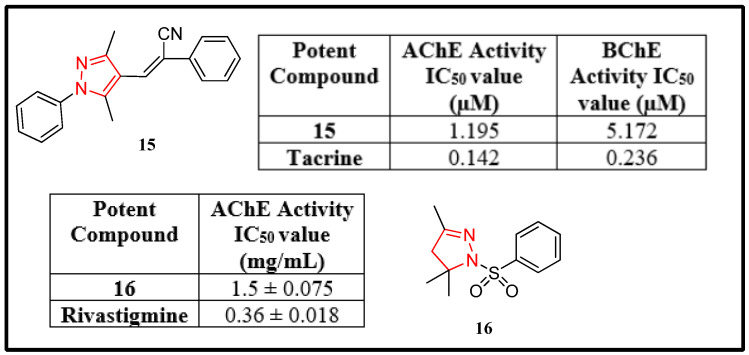
Some active pyrazole derivatives of compounds **15** and **16** are potent inhibitors of AChE and BChE [70,89].

Biçer has synthesized a series of 2,3-disubstituted heteroaryl acrylonitrile derivatives with IC_50_ values ranging from 1155.105 to 1.195 μM for AChE and 346.528–5.172 μM for BChE, and among them, compound **15** demonstrated the best AChE inhibition (IC_50_: 1.195 μM) and BChE inhibition (IC_50_: 5.172 μM) (Figure 14) [89]. Docking experiments indicate that compound **15** exhibits a superior binding affinity (−7.806 and −7.417 kcal/mol) in its interactions with 4EY7 (AChE) and 4BDS (BChE). Compound **15** demonstrated π stacking and hydrophobic interactions with target proteins 4EY7 and 4BDS. The amino acids are Tyr332, Tyr69, Leu73, Trp277, Tyr328, Phe329, and Tyr332, respectively. Also, it demonstrated π-stacking interactions with target protein 4BDS and amino acids Trp228 and Trp79, as well as hydrophobic interactions with protein 4BDS and amino acids Trp279, Thr117, Leu283, and Phe393. Additionally, compound **15** establishes a hydrogen bond with the Asp67 backbone residues of its target protein, 4BDS. In another study by Messaad et al., compound **16** (3,5,5-trimethyl-1-[(4-methylphenyl)sulfonyl]-4,5-dihydro-1H-pyrazole) demonstrated good inhibitory activity against AChE. This activity is similar to that of rivastagmine, with IC_50_ values of 1.5 ± 0.075 mg/mL (*p* < 0.05) and 0.36 ± 0.018 mg/mL (*p* < 0.05), respectively (Figure 14) [70].

**Figure 15:**
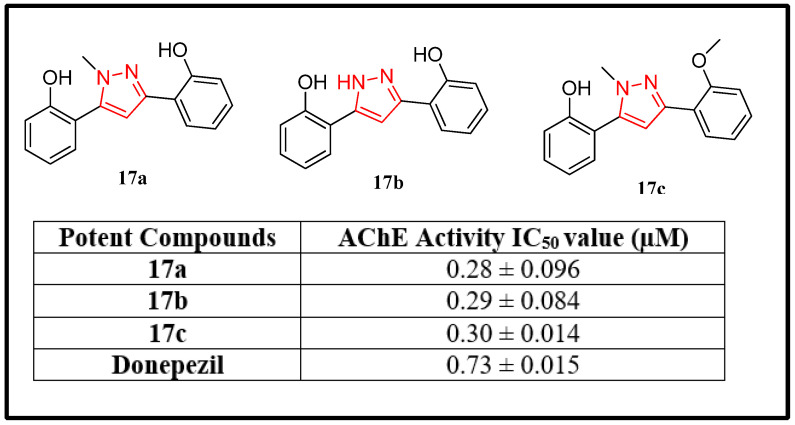
Some active diarylpyrazole derivatives of compounds **17a**, **17b,** and **17c** are potent AChE inhibitors [90].

**Figure 16:**
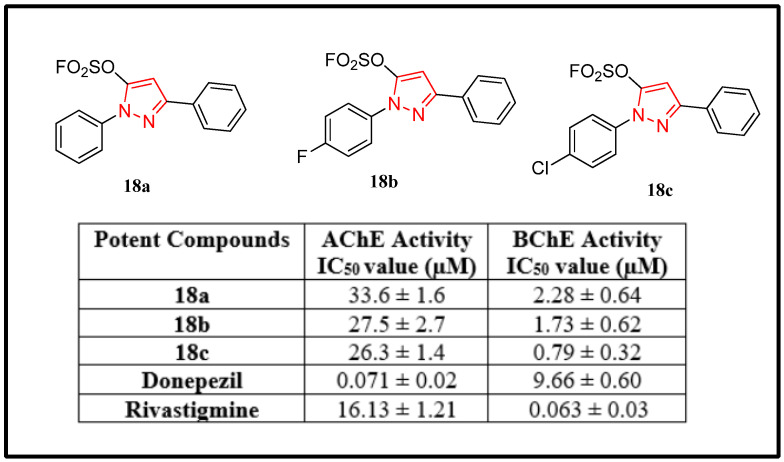
Fluorosulfate-containing pyrazole derivatives of compounds **18a**, **18b**, and **18c** as potent AChE and BChE inhibitors [91].

In a separate study by Zia and colleagues [90], a variety of diarylpyrazole derivatives were systematically synthesized. Among these compounds, some showed highly potent activity against AChE (Figure 15). Specifically, compounds **17a**, **17b**, and **17c** exhibited superior activity compared with the standard drug donepezil (more than twofold inhibitory activity than the reference drug), with IC_50_ values of 0.28 ± 0.096, 0.29 ± 0.084, and 0.30 ± 0.014 µg/mL at the respective concentrations. Also, molecular docking studies were applied to these compounds for AChE (PDB: 4M0E), and −9.0, −9.4, and −8.4 kcal/mol were obtained, respectively. Research conducted by Li et al. [91] discovered that pyrazole-5-fluorosulfates are effective inhibitors of BChE. Following the completion of a comprehensive investigation, several compounds were examined. As a consequence, the effects on the activity of BChE were demonstrated for 1-, 3-, 4-substituted, and 5-fluorosulfate of the pyrazole ring. Specifically, compounds **18a**, **18b**, and **18c** exhibited significant inhibitory properties against BChE, as indicated by their respective IC_50_ values of 2.28 ± 0.64, 1.73 ± 0.62, and 0.79 ± 0.32 μM, respectively. This contrasts with the standard drug donepezil, which exhibited an IC_50_ of 9.66 ± 0.60 μM, as highlighted in Figure 16.

**Figure 17:**
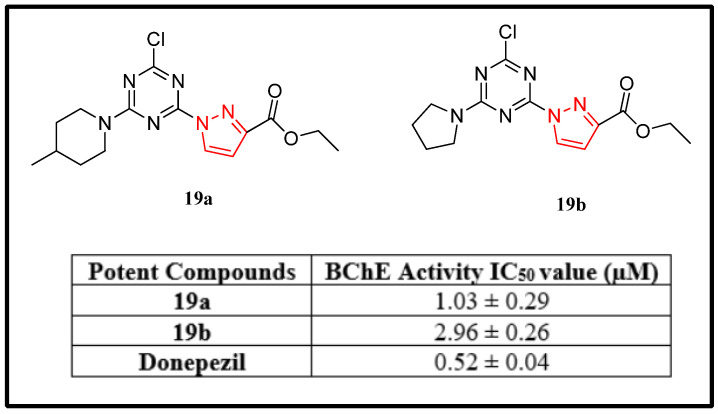
Pyrazole-1,3,5-triazine hybrid molecules of compounds **19a** and **19b** as potent BChE inhibitors [78].

Xue et al. developed and synthesized a series of thirteen pyrazole-1,3,5-triazine hybrid molecules, evaluating their efficacy as cholinesterase inhibitors [78]. Among the evaluated compounds, compound **19a** (ethyl 1-(4-chloro-6-(4-methylpiperidin-1-yl)-1,3,5-triazin-2-yl)-1H-pyrazole-3-carboxylate) had the highest efficiency in inhibiting BChE, with an IC_50_ value of 1.03 ± 0.29 µM, equivalent to the standard pharmaceutical drug donepezil (IC_50_ = 0.52 ± 0.04 µM) (Figure 17). Molecular docking investigations revealed persistent contact between compound **19a** and critical amino acids in both the CAS and PAS of BChE (PDB: 4TPK) with a binding energy of −9.67 kcal/mol. Molecular dynamics simulations demonstrate that compound **19a** can stably attach to BChE. Another active molecule from the same series was compound **19b**, exhibiting an IC_50_ value of 2.96 ± 0.26 µM (Figure 17). As potent, selective BChE inhibitors, these compounds may serve as promising lead molecules for Alzheimer’s disease research.

**Figure 18:**
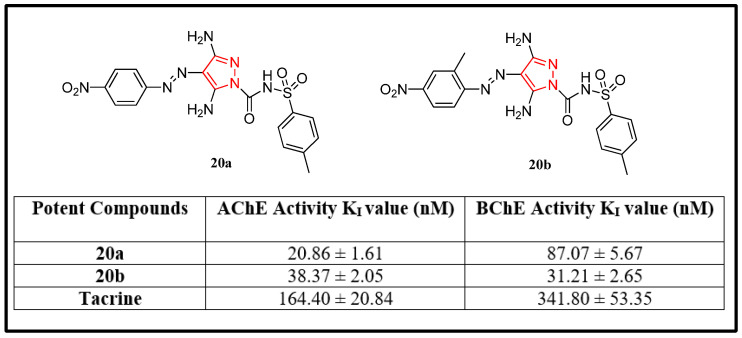
4-Arylazo-3,5-diamino-N-tosyl-1H-pyrazole-1-carboxamides of compounds **20a** and **20b** as potent AChE and BChE inhibitors [92].

A series of thirteen 4-arylazo-3,5-diamino-N-tosyl-1H-pyrazole-1- carboxamides was synthesized and evaluated against AChE and BChE in the most recent work by Akocak and colleagues [92]. Compared with the standard drug tacrine, all molecules have been demonstrated to be effective in the nanomolar range. Compounds **20a** and **20b**, which are among these chemicals, exhibited remarkable inhibitory characteristics, as evidenced by their K_I_ values of 20.86 ± 1.61 and 38.37 ± 2.05 nM, respectively, against AChE. However, the K_I_ values for BChE were 87.07 ± 5.67 and 31.21 ± 2.65 nM (Figure 18) [92].

**Figure 19:**
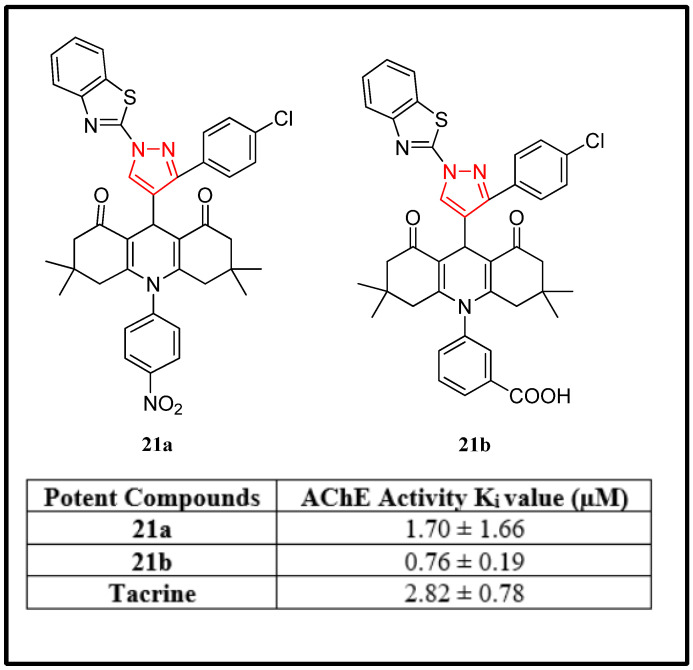
Pyrazole, acridine, and benzothiazole groups containing compounds **21a** and **21b** as potent AChE inhibitors [93].

Elmusa et al. developed and synthesized a variety of compounds comprising pyrazole, acridine, and benzothiazole groups as effective AChE inhibitors [93]. In vitro tests of AChE enzyme activity revealed that compounds **21a** (1.70 ± 1.66 μM) and **21b** (0.76 ± 0.19 μM) exhibited the most potent inhibition, as shown by their minimal K_i_ values (Figure 19). Molecular docking experiments demonstrated these processes with binding energies of −10.3 and −106.6 kcal/mol. The molecular docking studies indicated that compound **21b** formed a carbon-hydrogen bond with the TYR:341 residue.

**Figure 20:**
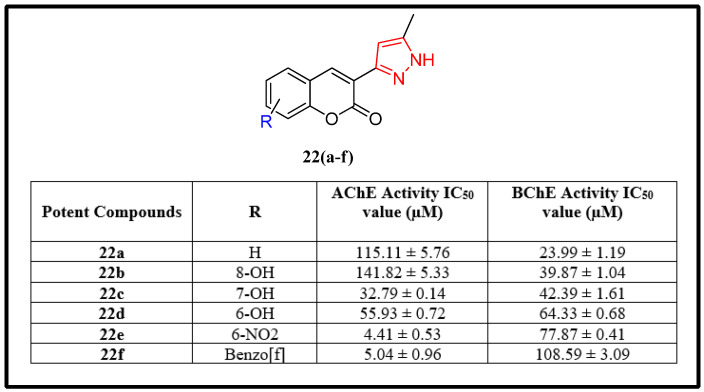
Pyrazole and coumarin-containing hybrid molecules; compounds **22**(**a**–**f**) as potent AChE and BChE inhibitors [94].

Recently, the integration of coumarin and pyrazole structures into a single molecule has emerged as a powerful multitask solution for the construction of cholinesterase-targeted inhibitory molecules. This inherent tendency of the coumarin moiety to interact with the PAS, together with the pyrazole ring’s ability to reach the catalytically active site, renders such hybrid systems highly attractive as dual inhibitors of both AChE and BChE. Hybrid compounds that are generated by the condensation of 3-acetoacetylcoumarin derivatives with hydrazines exhibit considerable AChE inhibition in nitro-substituted analogs. In contrast, hydroxyl-functionalized derivatives exhibit a strong level of selectivity to BChE. The growth of π-conjugation and the presence of electron-withdrawing functional groups are the main factors that are considered in this structural class and which enhance the binding affinity of AChE. Both molecular docking and binding-mode studies suggest that the coumarin’s main ring primarily occupies the PAS plane. In contrast, the pyrazole ring targets the hydrophobic sites of the catalytic site, thereby contributing to overall inhibitory potency. Toumani et al. synthesized a novel series of hybrid compounds using pyrazole and coumarin as inhibitors of AChE and BChE (Figure 20) [94]. In vitro tests demonstrated that compounds **22e** and **22f** had the highest efficacy, displaying significant AChE inhibitory activities with IC_50_ values of 4.41 ± 0.53 and 5.04 ± 0.96 µg/mL, respectively. Compounds **22a** and **22b** were identified as the most effective BChE inhibitors, exhibiting IC_50_ values comparable to those of standard pharmaceuticals (Figure 20) [94]. The docking investigations reveal that the ligand–protein complex in the active site has been stabilized by hydrophobic interactions, π-π stacking, and hydrogen bonding. In silico ADME analyses have shown that these substances had a favorable pharmacokinetic profile. The docking investigation of compound **22e** with the AChE enzyme demonstrated that the PAS is filled by the ligand. In silico toxicity models suggested that these molecules are likely to exhibit favorable oral bioavailability. Therefore, it was determined that the hybrid of pyrazole and coumarin scaffolds might be considered a promising anticholinesterase therapeutic agent and lead molecule for the development of Alzheimer’s disease drugs.

**Figure 21:**
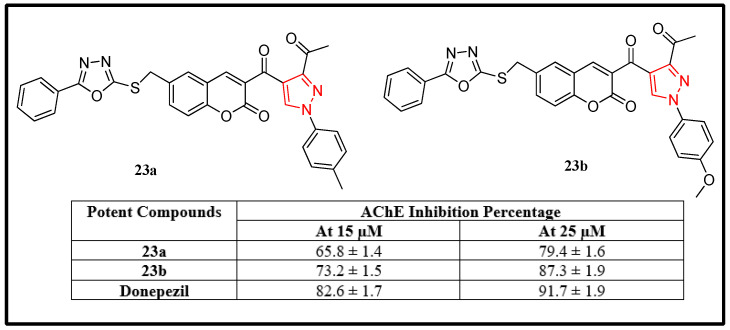
Coumarines attached to 1,3,4-oxadiazole and pyrazole unit compounds **23a** and **23b** as potent AChE inhibitors [95].

In a study by Ahmed et al., a series of new coumarines bearing 1,3,4-oxadiazole and/or pyrazole units were successfully synthesized, which are potent AChE inhibitors. Among these derivative compounds, **23a** and **23b** showed the highest inhibition percentages, with 79.4 ± 1.6 and 87.3 ± 1.9 at 25 µM, respectively (Figure 21) [95].

**Figure 22:**
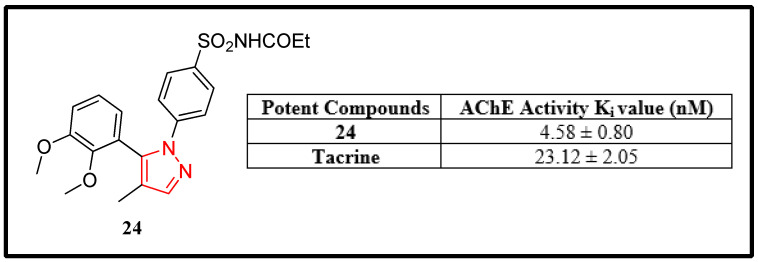
Celecoxib derivative including pyrazole-linked sulfonamide; compound **24** as potent AChE inhibitors [96].

Gerni et al. developed and evaluated celecoxib derivatives, including pyrazole-linked sulfonamide, against AChE [96]. Compound **24**, characterized by the presence of 2,3-dimethoxyphenyl groups and exhibiting the most potent inhibitory properties (K_i_ value of 4.58 ± 0.80 nM), demonstrated greater inhibition of AChE than the reference drug, tacrine (K_i_ value of 5.37 ± 1.13 nM) (Figure 22). A molecular docking analysis of compound **24** was conducted on the 3D crystallographic structure of AChE, revealing optimal binding poses with a binding energy of −9.2 kcal/mol. In the docking investigation conducted with eeAChE of compound **24**, it was noted that the compound interacted with the PAS and mid-gorge areas. In the PAS regions, the sulfonamide group established a standard hydrogen bond with Tyr124 and a π-sulfur interaction with Tyr314. The benzene ring linked to the sulfonamide group generated a π-π stacking interaction with Tyr341 and a π-π T-shaped interaction with Trp286. The 2,3-dimethoxy phenyl ring coupled to the pyrazole ring developed a π-π T-shaped interaction with Trp286 and a π-alkyl interaction with Tyr341. Following interactions within the mid-gorge, the pyrazole ring generated an amide-π stacking interaction with Ser293, while the benzene ring linked to the sulfonamide group produced a T-shaped connection with Phe297.

**Figure 23:**
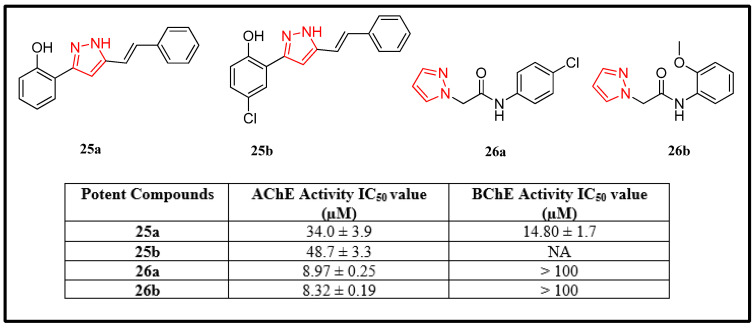
Pyrazole analogs of diarylpentanoids and phenylacetamide derivatives bearing 1H-pyrazole molecules (compounds **25**(**a**,**b**) and **26**(**a**,**b**)) as potent AChE and BChE inhibitors [97,98].

In a study conducted by Mohd Faudzi and colleagues, the effects of potential cholinesterase inhibitors with nitric oxide (NO) releasing properties on AChE and BuChE were investigated using in silico approaches (Figure 23) [97]. The binding affinities of various small molecules containing NO-donor functional groups for cholinesterase enzymes were evaluated using molecular docking. The results revealed that some compounds exhibited high affinity for the active site of AChE, with binding energies below −10.0 kcal/mol, while forming weaker but significant interactions with BChE. Docking analyses showed that π-π interactions formed with critical amino acids, particularly Trp86, Tyr337, Tyr341, and His447, played an important role in determining inhibitor activity [97].

In another study by Tarıkoğulları et al., the cholinesterase inhibitory potential of N-phenylacetamide derivatives containing pyrazole or 1,2,4-triazole rings was investigated (Figure 23) [98]. The structures of a total of 12 compounds obtained by a two-step synthesis strategy were verified using FT-IR, ^1^H-NMR, and HRMS techniques. Biological evaluation revealed that all compounds exhibited moderate but highly selective inhibitory activity against AChE. IC_50_ values for AChE ranged from 6.68 to 15.61 µM, while IC_50_ values for BuChE were reported to be above 100 µM. Structure–activity relationship analyses showed that the methoxy group in the phenyl ring increased the inhibitory activity of AChE, especially in 1,2,4-triazole derivatives. At the same time, chloride substitution was more advantageous in pyrazole derivatives. These findings reveal that the electronic and steric properties of substituents on the aromatic ring play a decisive role in enzyme inhibition [98].

Overall, it is clear that aromatic ring conjugation is one of the key structural parameters determining the enzyme selectivity of pyrazole-based cholinesterase inhibitors. Extending aromatic systems enhances selectivity, particularly by increasing hydrophobic and π-π stacking interactions within the large binding pocket of BChE. Conversely, more compact and stable conjugated systems provide better compatibility with the AChE active site, enabling the development of potent AChE inhibitors. Therefore, rational design of aromatic conjugation is considered a critical strategy in the development of novel pyrazole-based cholinesterase inhibitors.

### 4.2. Bis-Pyrazole Derivatives as Cholinesterase Inhibitors

A combination of two pyrazole pharmacophores in a single molecular architecture is a highly effective medicinal chemistry strategy that can be used to maximize the biological activity and molecular recognition [99,100]. The bis-pyrazole derivatives are more rigid, have larger surface areas, enhanced electronic characteristics, and higher conformational flexibility than their mono-pyrazole counterparts, which provide better opportunities for binding to multiple sites in the biological target [58,101]. These structural properties promote a higher hydrogen-bonding network, hydrophobic interactions, and π-π stacking with the amino acid residues, which typically leads to better binding affinity, selectivity, and pharmacological effects. Furthermore, the two pyrazole rings offer many possibilities for structural diversification through functionalization of the pyrazole core or by changing the linker between these two rings, allowing the rational design of compounds with different physicochemical and pharmacokinetic properties [102,103]. In the past few years, bis-pyrazole derivatives have become very attractive scaffolds for the design of new drugs for neurodegenerative diseases, such as Alzheimer’s disease (AD), which is one of the most prevalent causes of dementia in the world. Although significant progress has been made in the knowledge of the molecular pathology of AD, available pharmacological therapy is still mainly symptomatic and offers only moderate cognitive improvements. Bis-pyrazole derivatives have been the subject of many studies due to their ability to interact with both the CAS and the PAS of AChE and BChE. This dual-site binding has not only been shown to improve enzyme inhibition but also to inhibit aggregation of amyloid-β via AChE, offering a further disease-modifying action in addition to symptomatic relief. Moreover, the bis-pyrazole structure has remarkable synthetic versatility and can serve as a useful scaffold to develop bifunctional ligands that could target several pathological pathways associated with AD in a single molecule. The bis-pyrazole-based molecules can be made by adding other pharmacophoric groups and thus can be used to provide antioxidant, metal chelating, anti-inflammatory, anti-amyloidogenic, and neuroprotective properties, in addition to cholinesterase inhibition. As a result, bis-pyrazole derivatives have been more recently recognized as interesting lead structures for next-generation anti-Alzheimer compounds showing better efficacy, selectivity, and safety (Table 4).

**Figure 24:**
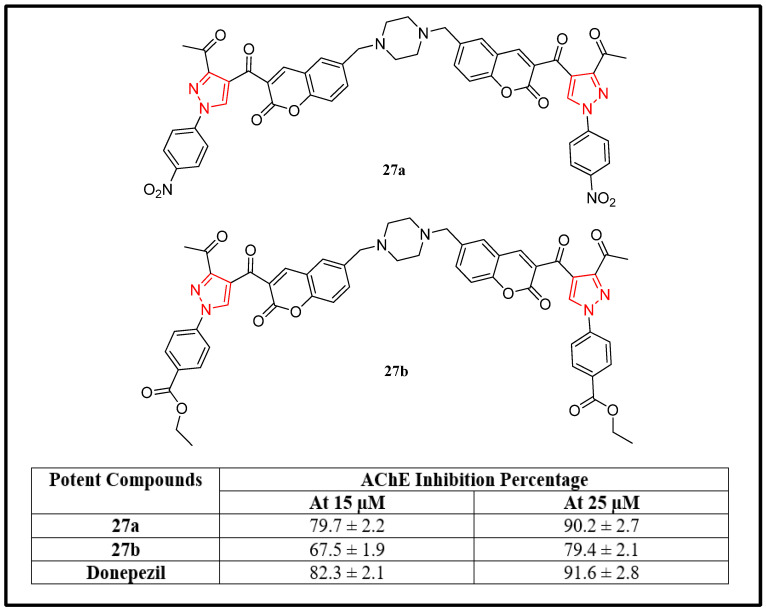
Piperazine-linked bis (coumarine) hybrids that are attached to pyrazole molecule compounds **27a** and **27b,** which are potent AChE inhibitors [104].

**Figure 25:**
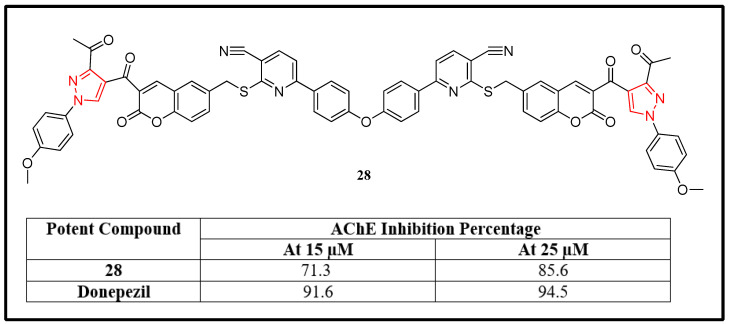
Bis (pyrazole) based on nicotinonitrile-coumarine derivative compound **28** as a potent AChE inhibitor [105].

Mekky et al. recently produced piperazine-linked bis (coumarine) hybrids that are coupled to pyrazole units [104]. These hybrids were designed to be effective AChE inhibitors, and their inhibitory activity was evaluated at 15 and 25 µM. At a concentration of 25 µM, two compounds, **27a** and **27b**, exhibited high inhibition, with inhibition percentages of 90.2 ± 2.7% and 79.4 ± 2.1%, respectively (Figure 24). Mekky et al. conducted another study in which they synthesized two series of bis (pyrazoles) based on nicotinonitrile-coumarine. Among these bis (pyrazoles), compound **28** demonstrated the highest level of AChE activity, with inhibition percentages of 71.3 (Figure 25) (Table 4) [105].

**Figure 26:**
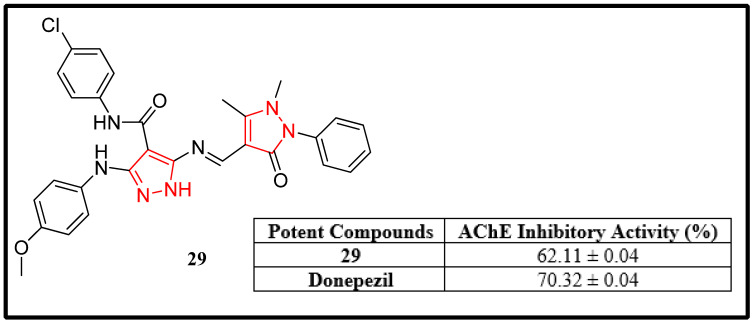
Pyrazole-based Schiff base compound **29** as a potent AChE inhibitor [106].

In a study by Naglah et al., a series of pyrazole-based Schiff bases were designed and synthesized as multi-target agents [106]. Among these molecules, compound **29** possessed a higher inhibitory effect on AChE (62.11 ± 0.04%) compared to the efficiency of donepezil (70.32 ± 0.04%) at the same concentrations (Figure 26).

### 4.3. Fused-Pyrazole Derivatives as Cholinesterase Inhibitors

**Figure 27:**
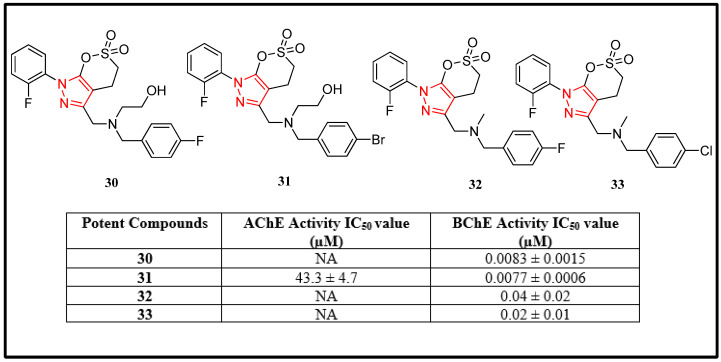
δ-sultone-fused pyrazoles (compounds **30**–**33**) as potent BChE inhibitors [107].

The electronic properties of substituents on the pyrazole ring play a critical role in both inhibitory activity and enzyme selectivity [107,109]. Electron-donating groups (e.g., -CH_3_, -OCH_3_) increase the number of π-electrons within the aromatic system, therefore raising aromatic and hydrophobic interactions within the active sites of both AChE and BChE. Gene expressions to optimize π-π stacking interactions with aromatic residues along the catalytic groove can greatly enhance binding affinity and total inhibitory effectiveness [110]. On the other hand, electron-withdrawing groups (e.g., -F, -Cl, -Br) normally increase lipophilicity and often confer more favorable interactions into the growing and increasingly hydrophobic binding pocket of BChE [109,110,111]. For example, the introduction of the electron-withdrawing substituents such as -F, -Cl, and -Br, as observed in compounds **21–24** with the groups 4-F, 4-Cl, and 4-Br, has also been reported to trigger changes in binding orientation and significantly change enzyme selectivity (Figure 27) (Table 4) [107]. These groups can alter the ligand’s binding orientation, thereby greatly affecting enzyme selectivity. In many cases, the higher the complementarity with the hydrophobic regions of the active site, the greater the inhibitory effect. The results of numerous studies collectively support the idea that the electron-withdrawing substituents significantly contribute to BChE selectivity. In contrast, electron-donating groups facilitate aromatic interactions with AChE, thereby enhancing affinity for this enzyme [107,109,110,111].

**Figure 28:**
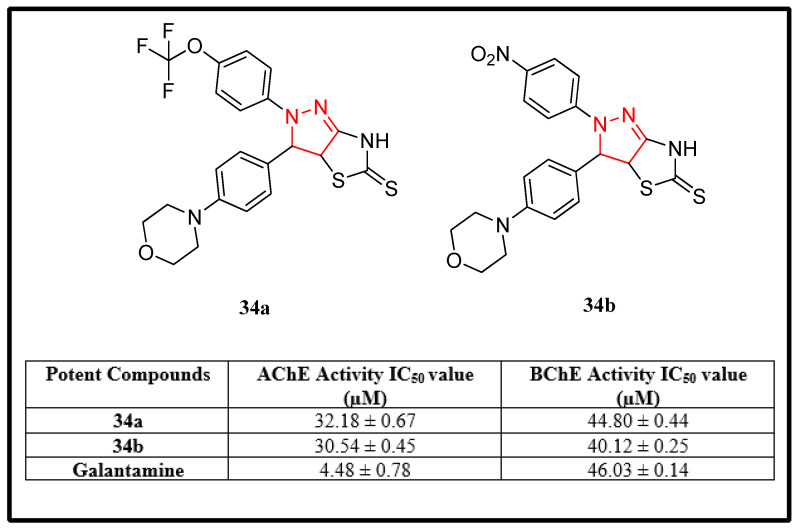
Some active pyrazolo derivatives of compounds **18a** and **18b** as potent AChE and BChE inhibitors [108].

Sicak et al. have developed and synthesized a series of 24 pyrazolo compounds that inhibit both AChE and BChE; these compounds are potent inhibitors. With IC_50_ values of 32.18 ± 0.67 and 30.54 ± 0.45 μM for AChE and 44.80 ± 0.44 and 40.12 ± 0.25 μM for BChE, respectively, compounds **34a** and **34b** were found to be the most powerful compounds among those that were produced (Figure 28). The compounds in question exhibited greater effectiveness as BChE inhibitors than galantamine, which has an IC_50_ of 46.03 ± 0.14 μM for BChE [108].

## 5. Trends in Artificial Intelligence and Machine Learning-Driven Drug Design

**Figure 29:**
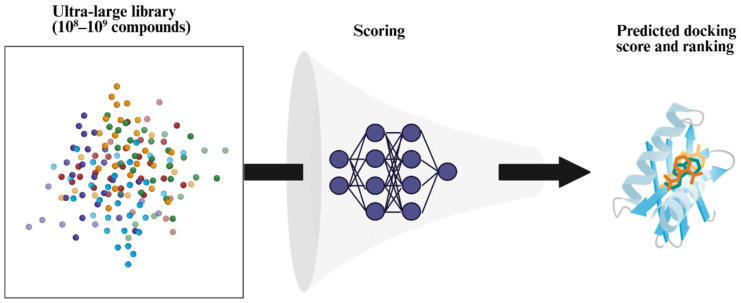
Schematic representation of DeepDocking.

Over the past two decades, major changes have transformed the drug development process, replacing experimental and high-throughput screening with data-driven, computationally informed approaches. More important steps towards this transformation have been the creation of the DeepDocking platform by Gentile and others (Figure 29) [112]. DeepDocking is a deep-learning-based method to scale an unlimited number of chemical reactions with binding energies that are orders of magnitude more cost-effective than solving Schrödinger equations and, at the same time, enables billion-scale virtual screening as a feasible and economical technology. In this framework, molecular binding affinities were extrapolated from two-dimensional representations, enabling highly precise predictions. With such skills, the model recognizes complex chemical characteristics such as the electronic nature of heteroaromatic rings, topological stability, and the ability to form 0-type interactions, which indicates an advanced rate of chemical pattern recognition brought about by artificial intelligence [112].

This technological breakthrough has significantly accelerated the use of machine learning in cholinesterase inhibitor identification and has led to a profound revolution in conventional screening models. Here, the creation of machine learning models based on large (thousands) datasets of AChE inhibitors by Herrera-Acevedo and colleagues served as an effective method to predict structural trends associated with cholinesterase binding and, importantly, these models predicted the association of cholinesterase with AChE with high predictive accuracy [113]. In their research, they tested several algorithms, including Random Forest (RF) and Gradient Boosted Trees (GBT), as well as Support Vector Machines (SVM), using the desktop set of descriptors, such as DRAGON and VolSurf+. Among them, RF and GBT models demonstrated greater effectiveness in separating active from inactive AChE inhibitors. Importantly, these frameworks did not rely solely on conventional physicochemical characteristics, such as lipophilicity, polar surface area, or hydrogen-bonding capacity. They could also determine more complex characteristics, such as aromatic density and models of the cation-pi interaction that characterize the CAS-PAS transition point of cholinesterases. Therefore, computational descriptions of the intricate biochemical properties of cholinesterase–ligand interactions were provided by machine learning methodologies, which serve as a formidable supplement to the traditional structure-based drug design systems [113].

Advancement of these methodological innovations was especially evident within the 2023–2025 period. Xiao and colleagues’ hybrid machine learning model proved capable of enabling artificial intelligence to analyze biologically diverse datasets for identifying cholinesterase inhibitors [114]. Their approach organized the 8614 non-peptidic inhibitors and 47 anti-AChE peptides seen in the literature into a single learning framework. Merging Morgan fingerprint representations with a Random Forest algorithm yielded an AUC of 0.94, indicating strong predictive performance. The most significant finding of the research was the correct identification of all active peptides in the external validation set with 100% accuracy. The identified discovery clearly indicates that artificial intelligence goes beyond the classification of small organic compounds; it can also continuously learn on hybrid datasets containing chemically diverse biotypes, such as peptides and non-peptidic ligands. The researchers emphasized that peptide sequences with strong π interactions with key aromatic residues in the catalytic cavity, including Trp84, Phe330, Tyr121, and so on, were typically characterized by the model as high-probability inhibitors. This observation implies that the learning process was not merely statistical but included biophysically significant interaction patterns, with a mechanistic emphasis on prediction [114]. These advances indicate that artificial intelligence techniques for cholinesterase inhibitor identification are applicable not only for speeding up screening campaigns but also positively influence the molecular quality of the final candidates. Deep learning models are useful because they can eliminate high-probability molecules in the large chemical space, thereby narrowing the search space, while ML-QSAR models can identify the structural variables that affect AChE binding. In conjunction with molecular docking, the predictions can then be biophysically validated, forming a synergistic pipeline linking statistical learning to structure-based reasoning. It therefore follows that in the initial stages of AI-inspired screening campaigns, one typically focuses on electrically rich, compact, and conformationally stable heterocyclic scaffolds. Many algorithms consistently identify pyrazole derivatives as the privileged structure. This is due to their favorable ring structure via aromatic stacking, the directed electrostatic distribution formed by the adjacent NN motif, and a balanced hydrogen-bond donor/acceptor profile, which allows them to integrate well into enzyme-binding sites. These properties of pyrazoles render them highly useful in AI-driven drug discovery and explain why they are often considered useful scaffolds in the development of cholinesterase inhibitors. The current literature clearly shows that artificial intelligence systems are no longer limited to traditional classification problems and docking scores; they are already being applied to the straightforward creation of novel molecular structures. Generation models based on variational autoencoders (VAEs), generative adversarial networks (GANs), and transformers can now be trained to sample cholinesterase-targeted pharmacophore distributions and sample new heterocyclic patients in a synthetically attainable chemical space. In these models, a trend is observed toward favoring five-membered, electron-dense, and highly planar structures, which are structurally similar to the natural characteristics of the pyrazole core. This unity produces a natural connection between modern generative algorithms and pyrazole chemistry: the structures displayed by the disorders of pharmacophoric patterns predicted by the models and the structural characteristics of the pyrazole ring are well suited to each other. The same electrical density, aromaticity, and geometric compactness that are attractive to medicinal chemists are also especially beneficial when it comes to using pyrazoles as scaffolds to create data-oriented molecules. In this regard, AI- and ML-guided drug design has ceased to be merely a statistical support tool and has become key technology for guiding chemical discovery in the field of cholinesterase inhibitors. Upon combining the virtual screening methodology with a wide range of structural descriptors of AChE proposed by Herrera-Acevedo [113], and the example of gaining a deeper insight into biological diversity learning provided by the hybrid models visualized by Xiao [114], one can conclude that modern approaches to artificial intelligence provide a high-resolution layer of computations that supply optimization information on pharmacophore-dense heterocyclic structures such as pyrazoles and enrich experimental chemistry significantly. Consequently, since 2020, studies on the development of cholinesterase inhibitors have increasingly relied on a pipeline in which AI-provided compounds are synthesized, biologically tested, and polymorphically raised and lowered in structure–activity relationships.

### 5.1. The Need for In Vivo Validation and Mechanistic Studies

**Figure 30:**
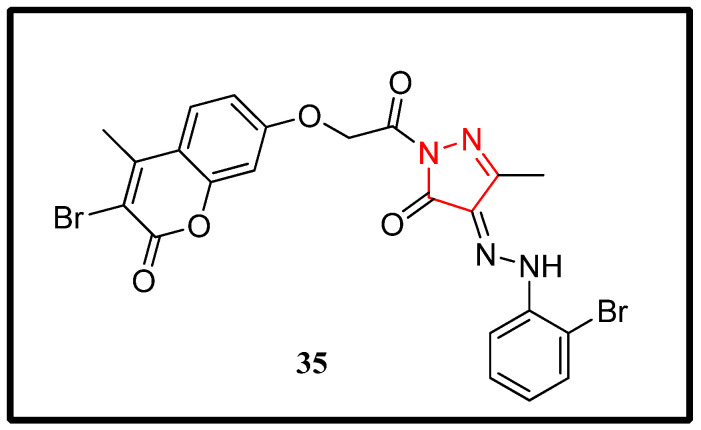
Chemical structure of compound **35**.

The derivatives of pyrazole are exceptionally critical in this new paradigm, as they serve as a chemically versatile scaffold and as preferred structures that are used in both predictive and creative operations, contrasting with advanced artificial intelligence engines. Even though the in vitro evidence supporting the therapeutic value of pyrazole-based cholinesterase inhibitors is significant, studies explaining their biological actions in the body remain limited. The in vivo models of Alzheimer’s disease are important for understanding the pharmacodynamic effects of pyrazole derivatives, as they will provide the overall evaluation of behavioral, biochemical, and histological phenotypes. Recent research is of special importance, as it suggests that pyrazole drugs may have a broader neuroprotective profile (not solely due to the traditional enzyme-inhibitory activity). A good example is the coumarin-pyrazole hybrid **35** (Figure 30), which has been heavily investigated with the help of the scopolamine-induced acute cholinergic impairment paradigm [115]. The results of the study indicate a dose-dependent reduction in performance in the elevated plus maze test in mice given **35,** suggesting significant improvement in learning and memory. These behavioral findings were strongly supported by chemical studies of brain tissue. Compared to the control (toxic) group, AChE activity had been reduced drastically, and the concentrations of malondialdehyde (MDA) and nitrite also declined significantly. At the same time, the role of endogenous antioxidant defenses (reduced glutathione (GSH), superoxide dismutase (SOD), and catalase (CAT)) was restored to approximately normal levels. These results indicate that compound **35** not only counters scopolamine-induced cholinergic impairment but also eliminates oxidative stress, thereby limiting cell damage and restoring redox balance in the brain. These findings were confirmed by histopathological and redundant data, which established that neuronal degeneration in the hippocampal CA1 region induced by scopolamine was greatly reduced after compound **35** treatment.

The evidence of behavioral improvement, biochemical rescue, and histological conservation is a rare and potent sign of the neuroprotective abilities of pyrazole derivatives in vivo. The stable molecular framework presented by Narayanan et al. [115] provides an important framework for the biomolecular targeting of the pyrazole-based inhibitors as multifunctional neuroprotective agents rather than acting as cholinesterase inhibitors per se. A more pathophysiologically relevant model of Alzheimer’s disease has been studied using the fluorosulfate-modified pyrazole derivative **18c** (Figure 16) following intracerebroventricular injection of Aβ_1–42_. The Morris water maze behavioral assessment showed that **18c** therapy produced a significant reduction in learning latencies and target quadrant occupancy duration, indicating significant improvement in spatial learning and memory. These behavioral benefits were also supported at the molecular level. The results of ELISA tests showed that **18c** reduced the cerebral Aβ load to a degree similar to that of Donepezil. PAMPA-BBB tests confirmed the ability of **19c** to pass through the blood–brain barrier via passive diffusion. Toxicological tests also yielded good results. The toxicity and hepatotoxicity tests under investigation have shown that no significant adverse effects occurred, even at an oral dose of 1 g/kg. These discoveries make **19c** one of the best-validated pyrazole-based candidates in in vivo Alzheimer’s disease studies to date [91]. Although there are compelling results in these two studies that pyrazole-based inhibitors can play beneficial roles in a biological system, including cognitive enhancement, cholinesterase inhibition, oxidative stress regulation, and ABB burden reduction, the present literature on using these reagents in vivo remains limited in scope and is thus largely reliant on acute experimental studies. Key translational aspects of the pathology, such as chronic models of Alzheimer’s disease, extensive pharmacokinetic and tissue distribution studies, extended toxicity studies, effects on tau pathology, as well as glial stimulation and neuroinflammatory markers, have not been well studied. Therefore, despite the high rate of current data, more needs to be done to improve the in vivo validation of pyrazole scaffolds to enable clinical application. This must include a longer research period, longer chronic disease models, and extended in-depth mechanistic studies to clearly elucidate their potential therapeutic value and safety profile.

### 5.2. Emerging Opportunities for Multi-Target and Personalized Pyrazole Inhibitors

**Figure 31:**
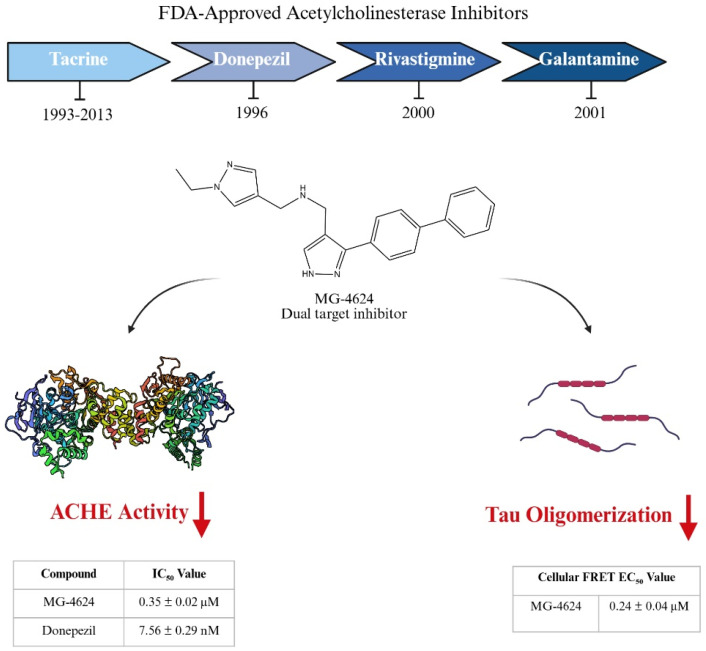
Dual-target inhibition of acetylcholinesterase activity and tau oligomerization by a pyrazole-based compound [116].

The growing importance of pyrazole analogs in neurodegenerative diseases is closely related to the complexity of Alzheimer’s disease. The combination of various interconnected pathogenic mechanisms, such as an imbalance between AChE and BChE activity, Aβ buildup, tau hyperphosphorylation, metal-ion-induced oxidative stress, and mitochondrial dysfunction, affects the course of the disease. Monolithic treatment methods inherently lack versatility because of the varying percentages and concentrations of distinct pathogenic elements among patients. The multi-target drug design has a strong basis with a pyrazole backbone. Functions such as the conjugated aromatic architecture, bis-hydrogen-bonding capability of the two adjacent nitrogen atoms, and chemical adaptability to have other pharmacophoric functions all combine to make pyrazole a natural multifunctional ensemble. A clear-cut illustration of this principle, through experiment, is provided by the biphenyl-pyrazole scaffold developed by Gabr and his colleagues [116]; the most active drug identified during high-throughput screening exhibited strong AChE inhibition, with an IC_50_ of 0.35 ± 0.02 μM, as determined by the Ellman assay (Figure 31). In particular, the same compound was shown to prevent tau oligomerization in the early phase by the status of Thioflavin-T fluorescence and transmission electron microscopy (TEM). In addition, it significantly reduced tau-induced cytotoxicity in SH-SY5Y neuronal cells, which transforms its twofold-target activity into an assessable biological benefit. The results show solid proof that a single pyrazole-based molecule can simultaneously stimulate cholinergic dysfunction and tau [116].

This type of pyrazoline derivative by Bajad and associates is yet another example of the ability of a distinctive chemical entity to attack a wide range of pathogenic nodes, whether in vivo or in vitro [117]. The strongest molecule in this series inhibited AChE with an IC_50_ of 2.89 μM and possessed greater efficacy towards BChE (IC_50_ = 0.151 μM). It also suppressed BACE-1 by about 36%, reduced Aβ_1–42_ aggregation by a significant margin, and exerted a cholinesterase inhibitory effect. The ability to permeabilize the central nervous system was confirmed by PAMPA-BBB experiments, which yielded a permeability coefficient (Pe) of 7.28 × 10^−6^ cm/s. The channeled chemical also improved Y-maze performance in behavioral tests using the scopolamine paradigm, indicating cognitive improvement. The in vivo studies found that malondialdehyde (MDA) levels decreased, whereas catalase (CAT) levels increased, highlighting its capacity to control oxidative stress through multiple biochemical pathways [117]. Together, these studies indicate a new category of pyrazole-based inhibitors, which are superior to traditional single-target models. They demonstrate that pyrazole frameworks can be developed with respect to the targeted sequences in cholinesterase, amyloidogenic pathways, tau aggregation, and oxidative stress. This plasticity increases their therapeutic capacity and helps design individual treatment regimens, in which molecular designs are tailored to the dominant pathogenic features of particular patients. The pyrazolopyridine hybrids reported by Waly and colleagues provide a good understanding of the high potential of the multi-target method [118]. The two most potent compounds showed considerable dual cholinesterase inhibition with IC_50_ values of approximately 0.16–0.17 μM for AChE and BChE, which is stronger than the rivastigmine inhibitory effect. In addition, the compounds acted as effective GSK-3β inhibitors (IC_50_ = 0.21–0.26 μM) and directly affected tau hyperphosphorylation. Furthermore, there was a 75–79% reduction in Aβ_1–42_ aggregation, and docking experiments confirmed their engagement with amino acid sequences in the tau core, providing a molecular basis for anti-tau activity. In addition, their ability to chelate Fe^2+^, Cu^2+^, and Zn^2+^ ions demonstrates another protective mechanism: preventing metal-induced reactive oxygen species (ROS) formation [118]. Additionally, the experimental outcomes are consistent with the natural chemical plasticity of the pyrazole scaffold, which can be structurally readjusted to accommodate a variety of target combinations. In this regard, the study undertaken by Pravin and Jozwiak provides a good conceptual framework [119]. Their analysis methodically demonstrated that linker length, the presence of electron-donating or electron-withdrawing functional groups, and other fusion methods determine the hybrid molecule’s bias toward AChE or metal-binding pathways. They clarified the molecular explanations of pyrazole-dependent hybrids’ capability to maintain balanced interactions with a wide range of biological targets simultaneously, as opposed to having a strong affinity to one target in particular, by integrating docking simulations, kinetic data, and structure–activity relationship analysis to explain their broad biological targeting capabilities [119].

Although the multi-target paradigm has a strong evidence base, clinical evidence suggests that only a slight benefit may be achievable, given the considerable heterogeneity in pathological missense mutations among patients. The experiences highlighted by Abduljawad and co-authors are quite relevant [120]. Disparities in Aβ accumulation, coping, inflammatory states, oxidative stress intensity, and genetic predisposition lead to a substantial variability in disease progression among individuals. Thus, it is unlikely that such a universal therapy profile is optimal. From this perspective, the modular features of the pyrazole scaffold present a considerable opportunity: its chemical versatility allows the use of a single core structure to inhibit AChE, decelerate tau, chelate a metal, or regulate Aβ, depending on the primary pathological features. This flexibility allows the introduction of a unified chemical system that will also support multi-target and personalized treatment strategies. The crucial question now is how the pyrazole-based system’s structural diversity can be translated into personalized therapies. The biological diversity of Alzheimer’s disease is too large to be described using a single mechanistic model. The balance between Aβ protein synthesis/clearance, the extent of cholinergic degeneration, the extent of tau disease, the measure of metal accumulation, and the size of neuroinflammation vary markedly across patients. This inconsistency clarifies the common inconsistencies regarding the therapeutic efficacy of conventional treatments. The comprehensive study conducted by Abduljawad et al. has shown that Aβ-induced inflammation and oxidative stress progress at different rates in patients, and that mutations in APP, PSEN1, and PSEN2 may shift the disease course toward alternative pathogenic pathways [103]. Notably, the variances can now be identified at an early stage through PET imaging, CSF Aβ/tau ratios, and peripheral biomarkers, implying that therapies should be tailored to individual illness profiles rather than following a standardized treatment plan [120].

Multi-target ligands based on pyrazole, in this case, offer a highly adaptable platform that can be chemically tailored to reflect patients’ pathology profiles. This results in a pyrazole backbone as a multi-target pharmacophore with high potential and is an appropriate starting point for the development of customized treatment regimens in Alzheimer’s disease.

## 6. Conclusions

Pyrazole derivatives are among the most popular molecular scaffolds for designing cholinesterase inhibitors due to their high structural versatility, desirable physicochemical properties, and ease of structural diversification. The heteroaromatic structure of the pyrazole core enables numerous substitution patterns, providing precise control over electronic distribution, steric effects, and lipophilicity. The tunability provided offers an important advantage in establishing strong and selective interactions with the CAS and PAS of cholinesterase enzymes. In this respect, the development of next-generation therapies to resolve cholinergic dysfunction has been directed towards drugs from the pyrazole family. Recent progress in medicinal chemistry has led to major improvements in the selectivity and efficacy of pyrazole-type cholinesterase inhibitors. Detailed SAR studies have demonstrated that minor structural changes in the core of the pyrazole or to the aromatic and aliphatic groups attached to the core can cause dramatic changes in the inhibitory activity of the pyrazole core-based soluble amine and/or carbonyl groups against AChE and BChE. The use of rational optimization processes has now become viable to obtain compounds with nanomolar inhibitory effects, excellent selectivity profiles, and improved drug-like characteristics. Such selectivity between AChE and BChE is of great therapeutic value because levels of expression and function of these enzymes in neurodegenerative diseases vary across different stages. Despite these promising advancements, the challenging process of converting chemically potent pyrazole molecules into viable drug development candidates still remains. Pharmacodynamics and pharmacokinetics, including metabolic stability, blood–brain barrier permeability, toxicological safety, and off-target effects, are critical considerations for assessing enzymatic potency. Next-generation studies should, therefore, go beyond performance distinctions and adopt a more cross-disciplinary perspective. Computational processes, including molecular docking, molecular dynamics simulations, and QSAR studies, are needed to elucidate binding forces, flexibility, and structure-based optimization strategies for an enzyme. These tools, when used in conjunction with advanced synthetic strategies and comprehensive biological analyses, such as enzyme kinetics, cell-based assays, and in vivo pharmacological studies, can significantly speed up lead compound identification and optimization. Overall, pyrazole-based scaffolds will continue to play a central role in cholinesterase inhibitor research by balancing key principles of medicinal chemistry with the more recent translational drug development methods. The rational synthesis, systematic incorporation of computational design, and multifaceted biological validation will be very important in countering the current limitations and in advancing pyrazole-related compounds as clinically important cholinesterase suppressors. By integrating various aspects, it allows for a deep understanding of enzyme–ligand interactions and can serve as an effective platform for identifying potential drugs to treat cholinesterase-related neurological disorders.

## Figures and Tables

**Table 1:** Comparison table of reaction conditions of some selected pyrazoles.

Product	Reaction Conditions	Yield	Time	Reference
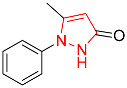	H_2_O, R.T., Microdroplets	47% Conversion	1–2 min	[43]
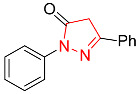	Acetic acid, Reflux	75%	24–36 h	[44]
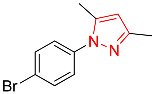	MeCN, R.T., Cu(NO_3_)_2_. H_2_O	87%	1 h	[45]
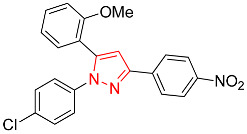	H_3_[PW_12_O_40_]/SiO_2_ 110 °C, M.W.	78%	30 min	[46]
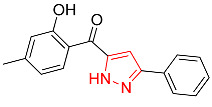	Reflux	90%	12 h	[47]
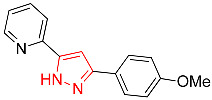	EtOH, Reflux	96%	Over Night	[48]

**Table 2:** Comparison table of reaction conditions of some selected pyrazoles synthesized by using multicomponent reaction procedures.

Product	Reaction Conditions	Yield	Time	Reference
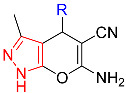	Et_3_N, EtOH	68–95%	60–270 min	[50]
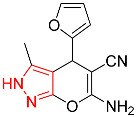	K-tBuO, MeOH, MW	90%	4–5 min	[51]
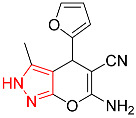	CSR, neat	90%	2–3 min	[52]
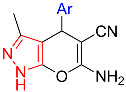	FSIPSS catalyst, EtOH, 40 °C, Ultrasonic	40–80%	3 min	[53]
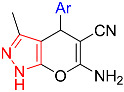	NEST, Solvent-free, R.T.	68–96%	10 min	[54]

**Table 3:** Comparison table of all mono-pyrazoles as potent cholinesterase inhibitors.

Compounds	AChE İnhibition	BChE İnhibition	Molecular Docking (kcal/mol)		Cytotoxicity	ADME Studies	Reference
			AChE	BChE			
**13a**	66.37 nM	83.93 nM	—	—	—	—	[87]
**13b**	70.83 nM	95.27 nM	—	—	—	—	[87]
**14a**	0.055 ± 0.143 µM	8.863 ± 0.22 µM	−12.191 (PDB: 1EVE)	—	HEK-293 N2a	Done	[88]
**14b**	0.017 ± 0.02 µM	6.331 ± 0.17 µM	−12.158 (PDB: 1EVE)	—	HEK-293 N2a	Done	[88]
**15**	1.195 µM	5.172 µM	−7.806 (PDB: 4EY7)	−7.417 (PDB: 4BDS)	—	Done	[89]
**16**	1.5 ± 0.075 µM	—	−7.5 (PDB: 4EY4)	—	—	—	[70]
**17a**	0.28 ± 0.096 µM	—	−9.0 (PDB: 4M9E)	—	—	—	[90]
**17b**	0.29 ± 0.084 µM	—	−9.4 (PDB: 4M9E)	—	—	—	[90]
**17c**	0.30 ± 0.015 µM	—	−8.4 (PDB: 4M9E)	—	—	—	[90]
**18a**	33.6 ± 1.6 µM	2.28 ± 0.64 µM	—	—	L02 HePG2	—	[91]
**18b**	27.5 ± 2.7 µM	1.73 ± 0.62 µM	—	—	L02 HePG2	—	[91]
**18c**	26.3 ± 1.4 µM	0.79 ± 0.32 µM	—	PDB: 6QAA	L02 HePG2	—	[91]
**19a**	—	1.03 ± 0.29 µM	—	−9.67 (PDB: 4TPK)	—	Done	[78]
**19b**	—	2.96 ± 0.26 µM	—	−8.81 (PDB: 4TPK)	—	Done	[78]
**20a**	20.86 ± 1.61 nM	87.07 ± 5.67 nM	−7.52 (PDB: 7XN1)	—	Beas-2B A549 MCF-7	Done	[92]
**20b**	38.37 ± 2.05 nM	31.21 ± 2.65 nM	—	−3.89 (PDB: 4BDS)	Beas-2B A549 MCF-7	Done	[92]
**21a**	1.70 ± 1.66 µM	—	−10.3 (PDB: 4EY7)	—	—	—	[93]
**21b**	0.76 ± 0.19 µM	—	−10.6 (PDB: 4EY7)	—	—	—	[93]
**22a**	115.11 ± 5.76 µM	23.99 ± 1.19 µM	−11.43 (PDB: 4EY7)	−12.23 (PDB: 5LKR)	—	Done	[94]
**22b**	141.82 ± 5.33 µM	39.87 ± 1.04 µM	−11.91 (PDB: 4EY7)	−11.80 (PDB: 5LKR)	—	Done	[94]
**22c**	32.79 ± 0.14 µM	42.39 ± 1.61 µM	−12.26 (PDB: 4EY7)	−11.05 (PDB: 5LKR)	—	Done	[94]
**22d**	55.93 ± 0.72 µM	64.33 ± 0.68 µM	−12.58 (PDB: 4EY7)	−11.01 (PDB: 5LKR)	—	Done	[94]
**22e**	4.41 ± 0.53 µM	77.87 ± 0.41 µM	−13.51 (PDB: 4EY7)	−10.87 (PDB: 5LKR)	—	Done	[94]
**22f**	5.04 ± 0.96 µM	108.59 ± 3.09 µM	−13.52 (PDB: 4EY7)	−10.33 (PDB: 5LKR)	—	Done	[94]
**23a**	79.4 ± 1.6 (% inhibition at 25 µM)	—	—	—	MCF-10A	—	[95]
**23b**	87.3 ± 1.9 (% inhibition at 25 µM)	—	—	—	MCF-10A	—	[95]
**24**	4.58 ± 0.80 µM	—	−8.10 (PDB: 1C2B)	—	—	—	[96]
**25a**	34.0 ± 3.9 µM	14.80 ± 1.7 µM	—	—	RAW264.7	Done	[97]
**25b**	48.7 ± 3.3 µM	NA	—	—	RAW264.7	Done	[97]
**26a**	8.97 ± 0.25 µM	>100	—	—	—	—	[98]
**26b**	8.32 ± 0.19 µM	>100	—	—	—	—	[98]

**Table 4:** Comparison table of all bis-pyrazoles and fused pyrazoles as potent cholinesterase inhibitors.

Compounds	AChE Inhibition	BChE Inhibition	Molecular Docking (kcal/mol)		Cytotoxicity	ADME Studies	Reference
			AChE	BChE			
**27a**	90.2 ± 2.7 (% inhibition at 25 µM)	—	—	—	MCF-10A	—	[104]
**27b**	79.4 ± 2.1 (% inhibition at 25 µM)	—	—	—	MCF-10A	—	[104]
**28**	85.6 (% inhibition at 50 µM)	—	—	—	—	—	[105]
**29**	62.11 ± 0.04 (% inhibition)	—	—	—	A549 Caco-2 WI-38	Done	[106]
**30**	NA	0.0083 ± 0.0015 µM	—	—	—	Done	[107]
**31**	43.3 ± 4.7 µM	0.0077 ± 0.0006 µM	—	PDB: 5LKR	HepG2 LO2	Done	[107]
**32**	NA	0.04 ± 0.02 µM	—	—	—	Done	[107]
**33**	NA	0.02 ± 0.01 µM	—	—	—	Done	[107]
**34a**	32.18 ± 0.67 µM	44.80 ± 0.44 µM	—	—	—	—	[108]
**34b**	30.54 ± 0.45 µM	40.12 ± 0.25 µM	—	—	—	—	[108]

## Data Availability

The original contributions presented in this study are included in the article. Further inquiries can be directed to the corresponding authors.

## References

[1] Lamptey R.N.L., Chaulagain B., Trivedi R., Gothwal A., Layek B., Singh J. (2022). A Review of the common neurodegenerative nisorders: Current therapeutic approaches and the potential role of nanotherapeutics. Int. J. Mol. Sci..

[2] Zhu X., Xu J.-B., Gao F., Wan L.-X. (2025). Advances in the structural modification of Alzheimer’s disease drug–Huperzine A. Bioorg. Chem..

[3] Mahajan G., Pandya K., Tripathi A., Kumar D. (2025). Integrating molecular imaging with near-infrared theranostics to improve early detection and therapy of Alzheimer’s disease. Bioorg. Chem..

[4] Ivanova A.V., Kutuzova A.D., Kuzmichev I.A., Abakumov M.A. (2025). Alzheimer’s Disease: From molecular mechanisms to mromising mherapeutic mtrategies. Int. J. Mol. Sci..

[5] Mieling M., Meier H., Bunzeck N. (2023). Structural degeneration of the nucleus basalis of Meynert in mild cognitive impairment and Alzheimer’s disease—Evidence from an MRI-based meta-analysis. Neurosci. Biobehav. Rev..

[6] Walczak-Nowicka Ł.J., Herbet M. (2021). Acetylcholinesterase inhibitors in the treatment of neurodegenerative diseases and the role of acetylcholinesterase in their pathogenesis. Int. J. Mol. Sci..

[7] Kabir M.T., Uddin M.S., Begum M.M., Thangapandiyan S., Rahman M.S., Aleya L., Mathew B., Ahmed M., Barreto G.E., Ashraf G.M. (2019). CholinesteraseiInhibitors for Alzheimer’s disease: Multitargeting strategy based on anti-Alzheimer’s drugs repositioning. Curr. Pharm. Des..

[8] Villeda-González J.D., Gómez-Olivares J.L., Baiza-Gutman L.A. (2024). New paradigms in the study of the cholinergic system and metabolic diseases: Acetyl-and-butyrylcholinesterase. J. Cell. Physiol..

[9] Mello-Hortega J.V., de Oliveira C.S., de Araujo V.S., Furtado-Alle L., Tureck L.V., Souza R.L.R. (2025). Cannabidiol and Alzheimer disease: A comprehensive review and in silico insights into molecular interactions. Eur. J. Neurosci..

[10] Akocak S., Boga M., Lolak N., Tuneg M., Sanku R.K.K. (2019). Design, synthesis and biological evaluation of 1,3-diaryltriazenesubstituted sulfonamides as antioxidant, acetylcholinesterase and butyrylcholinesterase inhibitors. J. Turk. Chem. Soc. Sect. A Chem..

[11] Djemil S., Sames A.M., Pak D.T.S. (2023). ACh transfers: Homeostatic plasticity of cholinergic synapses. Cell. Mol. Neurobiol..

[12] Hajimohammadi S., Lockridge O., Masson P. (2025). New views on physiological functions and regulation of butyrylcholinesterase and potential therapeutic interventions. Front. Mol. Biosci..

[13] Türkeş C., Lolak N., Duran H.E., Yapar G., Akocak S. (2025). Molecular and structural characterization of ureido-benzenesulfonamides as dual inhibitors of aldose reductase and cholinesterases. Arch. Biochem. Biophys..

[14] Novales N.A., Schwans J.P. (2022). Comparing the effects of organic cosolvents on acetylcholinesterase and butyrylcholinesterase activity. Anal. Biochem..

[15] Xing S., Li Q., Xiong B., Chen Y., Feng F., Liu W., Sun H. (2021). Structure and therapeutic uses of butyrylcholinesterase: Application in detoxification, Alzheimer’s disease, and fat metabolism. Med. Res. Rev..

[16] Tok F., Özer F.A., Kuzu Z.S., Sıcak Y., Kemaloğlu F., Baday S., Sungur F.A., Öztürk M. (2026). Synthesis, biological evaluation and molecular docking of novel 2-pyrazoline-1-carboxamides as anti-Alzheimer agents. Future Med. Chem..

[17] Bilen E., Özmen Ü.Ö., Çete S., Alyar S., Yaşar A. (2022). Bioactive sulfonyl hydrazones with alkyl derivative: Characterization, ADME properties, molecular docking studies and investigation of inhibition on choline esterase enzymes for the diagnosis of Alzheimer’s disease. Chem. Biol. Interact..

[18] Tekeli T., Lolak N., Tekeli Y., Bozgeyik E., Anakok D.A., Çete S., Gülçin I., Akocak S. (2024). Bis-ureido-substituted benzenesulfonamides: Evaluation of their antibacterial, anticholinesterase, and cytotoxicity properties. ChemistrySelect.

[19] Pepeu G., Giovannini M.G. (2010). Cholinesterase inhibitors and memory. Chem. Biol. Interact..

[20] Veselov I.M., Vinogradova D.V., Maltsev A.V., Shevtsov P.N., Spirkova E.A., Bachurin S.O., Shevtsova E.F. (2023). Mitochondria and oxidative stress as a link between Alzheimer’s disease and diabetes Mellitus. Int. J. Mol. Sci..

[21] Koca M., Güller U., Güller P., Dağalan Z., Nişancı B. (2022). Design and synthesis of novel dual cholinesterase inhibitors: In vitro inhibition studies supported with molecular docking. Chem. Biodivers..

[22] Kumar B., Thakur A., Dwivedi A.R., Kumar R., Kumar V. (2022). Multi-target-directed ligands as an effective strategy for the treatment of Alzheimer’s disease. Curr. Med. Chem..

[23] Ibrahimova N., Çete S., Anakök D.A., Özmen Ü.Ö., Gündüzalp A.B., Öztürk A., Aydemir I. (2025). Synthesis of new sulfa drugs containing FDA-approved sulfa pyridine: Evaluation of cholinesterase inhibition, antimicrobial, antibiofilm, anticancer, and antioxidant activities, along with theoretical calculation and molecular docking study. J. Mol. Struct..

[24] Almaghrabi M. (2025). Multitarget-directed ligands for Alzheimer’s disease: Recent novel MTDLs and mechanistic insights. Pharmaceuticals.

[25] Rossi M., Freschi M., de Camargo Nascente L., Salerno A., de Melo Viana Teixeira S., Nachon F., Chantegreil F., Soukup O., Prchal L., Malaguti M. (2021). Sustainable drug discovery of multi-target-directed ligands for Alzheimer’s disease. J. Med. Chem..

[26] Ghazarian A.L., Haim T., Sauma S., Katiyar P. (2022). National institute on aging seed funding enables Alzheimer’s disease startups to reach key value inflection points. Alzheimer’s Dement..

[27] Bennani F.E., Doudach L., Cherrah Y., Ramli Y., Karrouchi K., Ansar M., Faouzi M.E.A. (2020). Overview of recent developments of pyrazole derivatives as an anticancer agent in different cell line. Bioorg. Chem..

[28] Ansari A., Ali A., Asif M., Shamsuzzaman S. (2017). Review: Biologically active pyrazole derivatives. New J. Chem..

[29] Akocak S., Lolak N., Ammara A., Gürbüz G., Bozgeyik I., Taşdemir D., Hashem H., Brase S., Supuran C.T. (2026). Investigating the inhibitory properties of diazo and pyrazole-carboxamide-linked benzenesulfonamides against carbonic anhydrase isoforms I, II, IX, and XII. ChemMedChem.

[30] Ebenezer O., Shapi M., Tuszynski J.A. (2022). A Review of the recent development in the synthesis and biological evaluations of pyrazole derivatives. Biomedicines.

[31] Zhang Y., Wu C., Zhang N., Fan R., Ye Y., Xu J. (2023). Recent advances in the development of pyrazole derivatives as anticancer agents. Int. J. Mol. Sci..

[32] Akocak S., Lolak N., Ammara A., Özensoy Güler Ö., Supuran C.T. (2025). Exploring the carbonic anhydrase activation properties of 4-arylazo-3,5-diamino-1H-pyrazoles against hCA I, II, IV, and VII isoenzymes. Curr. Top. Med. Chem..

[33] Ríos M.C., Portilla J. (2022). Recent advances in synthesis and properties of pyrazoles. Chemistry.

[34] Rusu A., Moga I.M., Uncu L., Hancu G. (2023). The role of five-membered heterocycles in the molecular structure of antibacterial drugs used in therapy. Pharmaceutics.

[35] Li G., Cheng Y., Han C., Song C., Huang N., Du Y. (2022). Pyrazole-containing pharmaceuticals: Target, pharmacological activity, and their SAR studies. RSC Med. Chem..

[36] Alam M.J., Alam O., Naim M.J., Nawaz F., Manaithiya A., Imran M., Thabet H.K., Alshehri S., Ghoneim M.M., Alam P. (2022). Recent advancement in drug design and discovery of pyrazole biomolecules as cancer and inflammation therapeutics. Molecules.

[37] Meanwell N.A. (2014). The influence of bioisosteres in drug design: Tactical applications to address developability problems. Top. Med. Chem..

[38] Sarwar T., Mustafa G., Zafar W., Hassan A.U., Sumrra S.H., Asif A. (2025). Pyrazoles a privileged scaffold in drug discovery—Synthetic strategies & exploration of pharmacological potential. J. Mol. Struct..

[39] Costa R.F., Turones L.C., Cavalcante K.V.N., Júnior I.A.R., Xavier C.H., Rosseto L.P., Napolitano H.B., da Silva P.F., Neto M.L.F., Galvão G.M. (2021). Heterocyclic compounds: Pharmacology of pyrazole analogs from rational structural considerations. Front. Pharmacol..

[40] El Fadili M., Ez-Zoubi A., Aloui M., Mujwar S., Abuelizz H.A., Elhalaoui M., Amin A. (2025). Design of novel pyrazole and benzofuran-based derivatives as potent acetylcholinesterase inhibitors for Alzheimer’s disease management. Front. Chem..

[41] Ran F., Dong L., Liu Y., Zhao G. (2024). Novel 1,3,4-trisubstituted pyrazolopyrimidine derivatives show potent antiproliferative activity in mantle cell lymphoma. Lett. Drug Des. Discov..

[42] Zhi Y., Li B., Yao C., Li H., Chen P., Bao J., Qin T., Wang Y., Lu T., Lu S. (2018). Discovery of the selective and efficacious inhibitors of FLT3 mutations. Eur. J. Med. Chem..

[43] Jana M., Unni K., Pradeep T., Cooks R.G. (2025). Accelerated synthesis of pyrazoles mediated by water microdroplets. ACS Sustain. Chem. Eng..

[44] Kumar V., Chang C.K., Tan K.P., Jung Y.S., Chen S.H., Cheng Y.S.E., Liang P.H. (2014). Identification, synthesis, and evaluation of new neuraminidase inhibitors. Org. Lett..

[45] Wang H., Sun X., Zhang S., Liu G., Wang C., Zhu L., Zhang H. (2018). Efficient copper-catalyzed synthesis of substituted pyrazoles at room temperature. Synlett.

[46] Zhang D., Ren L., Liu A., Li W., Liu Y., Gu Q. (2022). One-pot solvent-free synthesis of 1,3,5-trisubstituted 1H-pyrazoles catalyzed by H_3_[PW_12_O_40_]/SiO_2_ under microwave irradiation. Monatsh. Cehm..

[47] Ahmed Mahmoud Abdel Reheim M., Saad Abdel Hafiz I., Adel Mohamed Sarhan A., Mohamed Reffat H. (2021). Synthesis and biological studies of some new pyrazole, dihydropyridinethione, pyrimidine, thiophene and 4H-pyran derivatives. Heterocycles.

[48] Javaid R., Rehman A.U., Ahmed M., Karouei M.H., Sayyadi N. (2022). Synthesis and photophysical investigations of pyridine-pyrazolate bound boron(III) diaryl complexes. Sci. Rep..

[49] Ahmad A., Rao S., Shetty N.S. (2023). Green multicomponent synthesis of pyrano [2,3-c]pyrazole derivatives: Current insights and future directions. RSC Adv..

[50] Shukla P., Sharma A., Anthal S., Kant R. (2015). Synthesis of functionalized pyrazolopyran derivatives: Comparison of two-step vs. one-step vs. microwave-assisted protocol and X-ray crystallographic analysis of 6-amino-1,4-dihydro-3-methyl-4-phenylpyrano [2,3-c]pyrazole-5-carbonitrile. Bull. Mater. Sci..

[51] Yallappa G.N., Dasappa N., Chandrashekhar U., Aruna G.L. (2022). One-pot multi component microwave assisted synthesis of 4H-pyrano [2, 3-c] pyrazoles in methanol and their antibacterial study. Lett. Appl. NanoBioSci..

[52] Gadkari Y.U., Hatvate N.T., Telvekar V.N. (2021). Concentrated solar radiation-assisted one-pot/multicomponent synthesis of pyranopyrazole derivatives under neat condition. Res. Chem. Intermed..

[53] Beiranvad M., Habibi D. (2022). Design, preparation and application of the semicarbazide-pyridoyl-sulfonic acid-based nanocatalyst for the synthesis of pyranopyrazoles. Sci. Rep..

[54] Dehghani Tafti A., Mirjalili B.B.F., Bamoniri A., Salehi N. (2021). Rapid four-component synthesis of dihydropyrano [2,3-c]pyrazoles using nano-eggshell/Ti(IV) as a highly compatible natural based catalyst. BMC Chem..

[55] Kumar K.S., Robert A.R., Rao A.V., Thainana S.K., Harikrishna S., Maddila S. (2024). A rapid, efficient microwave-assisted synthesis of novel bis-pyrazole analogues using non-toxic and cost-effective catalyst under green solvent medium. Chem. Data Collect..

[56] Mohammed D.A., Abdullah M.N. (2026). Green one-pot protocol for the synthesis of novel Pyrazole Derivatives by DES catalyst: In vitro biological application and comprehensive computational studies. J. Mol. Struct..

[57] Świętczak E., Rachwalski M., Pieczonka A.M. (2025). Eco-friendly methods for the synthesis of N-acyl pyrazole derivatives with luminescent properties. RSC Adv..

[58] Merzouki O., Arrousse N., Ech-chihbi E., Alanazi A.S., Mabrouk E.H., Hefnawy M., El Moussaoui A., Touijer H., El Barnossi A., Taleb M. (2025). Environmentally friendly synthesis of new mono- and bis-pyrazole derivatives; In vitro antimicrobial, antifungal, and antioxidant activity; and in silico studies: DFT, ADMETox, and molecular docking. Pharmaceuticals.

[59] De Boer D., Nguyen N., Mao J., Moore J., Sorin E.J. (2021). A comprehensive review of cholinesterase modeling and simulation. Biomolecules.

[60] Zhou Y., Wang S., Zhang Y. (2010). Catalytic reaction mechanism of acetylcholinesterase determined by born-oppenheimer Ab initio QM/MM molecular dynamics simulations. J. Phys. Chem. B.

[61] Hung L.W., Sanbonmatsu K.Y., Williams R.F., Chen J.C.H. (2025). Acetylcholinesterase: Structure, dynamics, and interactions with organophosphorus compounds. Protein Sci..

[62] Dvir H., Silman I., Harel M., Rosenberry T.L., Sussman J.L. (2010). Acetylcholinesterase: From 3D structure to function. Chem. Biol. Interact..

[63] Xu Y., Cheng S., Sussman J.L., Silman I., Jiang H. (2017). Computational studies on acetylcholinesterases. Molecules.

[64] Grabowska W., Bijak M., Szelenberger R., Gorniak L., Podogrocki M., Harmata P., Cichon N. (2025). Acetylcholinesterase as a Multifunctional Target in Amyloid-Driven Neurodegeneration: From Dual-Site Inhibitors to Anti-Agregation Strategies. Int. J. Mol. Sci..

[65] Cheung J., Gary E.N., Shiomi K., Rosenberry T.L. (2013). Structures of human acetylcholinesterase bound to dihydrotanshinone i and territrem B show peripheral site flexibility. ACS Med. Chem. Lett..

[66] Shrestha S., Seong S.H., Paudel P., Jung H.A., Choi J.S. (2018). Structure related inhibition of enzyme systems in cholinesterases and BACE1 in vitro by naturally occurring naphthopyrone and its glycosides isolated from cassia obtusifolia. Molecules.

[67] Sungthong B., Sithon K., Panyatip P., Tadtong S., Nunthaboot N., Puthongking P. (2022). Quantitative analysis and in silico molecular docking screening for acetylcholinesterase inhibitor and ADME prediction of coumarins and carbazole alkaloids from Clausena harmandiana. Rec. Nat. Prod..

[68] Peitzika S.C., Pontiki E. (2023). A review on recent approaches on molecular docking studies of novel compounds targeting acetylcholinesterase in Alzheimer disease. Molecules.

[69] Cetin A., Oguz E., Türkan F. (2022). In silico and in vitro analysis of acetylcholinesterase and glutathione S-transferase enzymes of substituted pyrazoles. Russ. J. Gen. Chem..

[70] Messaad M., Dhouib I., Abdelhedi M., Khemakhem B. (2022). Synthesis, bioassay and molecular docking of novel pyrazole and pyrazolone derivatives as acetylcholinesterase inhibitors. J. Mol. Struct..

[71] Abdelwahab H.E., Ibrahim H.Z., Omar A.Z. (2023). Design, Synthesis, DFT, molecular docking, and biological evaluation of pyrazole derivatives as potent acetyl cholinestrease inhibitors. J. Mol. Struct..

[72] Jaman S., Tasmi S.F., Shahriar I., Halim M.A. (2025). Histidine focused covalent inhibitors targeting acetylcholinesterase: A computational pipeline for multisite therapeutic discovery in Alzheimer’s disease. ACS Chem. Neurosci..

[73] Son M., Park C., Rampogu S., Zeb A., Lee K.W. (2019). Discovery of novel acetylcholinesterase inhibitors as potential candidates for the treatment of alzheimer’s disease. Int. J. Mol. Sci..

[74] Merzoug A., Boucherit H., Khaled R., Chefiri A., Chikhi A., Bensegueni A. (2021). Molecular docking study of the acetylcholinesterase inhibition. Curr. Issues Pharm. Med. Sci..

[75] Van Belle D., De Maria L., Iurcu G., Wodak S.J. (2000). Pathways of ligand clearance in acetylcholinesterase by multiple copy sampling. J. Mol. Biol..

[76] Simeon S., Anuwongcharoen N., Shoombuatong W., Malik A.A., Prachayasittikul V., Wikberg J.E.S., Nantasenamat C. (2016). Probing the origins of human acetylcholinesterase inhibition via QSAR modeling and molecular docking. PeerJ.

[77] El Alaouy M.A., Alaqarbeh M., Ouabane M., Zaki H., ElBouhi M., Badaoui H., Moukhliss Y., Sbai A., Maghat H., Lakhlifi T. (2024). Computational prediction of 3,5-diaryl-1H-pyrazole and spiropyrazolines derivatives as potential acetylcholinesterase inhibitors for alzheimer disease treatment by 3D-QSAR, molecular docking, molecular dynamics simulation, and ADME-Tox. J. Biomol. Struct. Dyn..

[78] Xue X.-Y., Wei M., Zhao Z., Xu L., Cao Y., Chen S., Hu P., Shi D.-H. (2025). Synthesis, biological activity, X-ray crystallographic, molecular docking and molecular dynamics simulation studies of pyrazole-1,3,5-triazine derivatives as potential butyrylcholinesterase inhibitors. J. Mol. Struct..

[79] Lolak N., Akocak S., Topal M., Koçyiğit Ü.M., Işık M., Türkeş C., Topal F., Durgun M., Beydemir Ş. (2025). Sulfonamide-bearing pyrazolone derivatives as multitarget therapeutic agents: Design, synthesis, characterization, biological evaluation, in silico ADME/T profiling and molecular docking study. Pharmacol. Res. Perspect..

[80] Lolak N., Boga M., Sonmez G.D., Tuneg M., Dogan A., Akocak S. (2022). In silico studies and DNA cleavage, antioxidant, acetylcholinesterase, and butyrylcholinesterase activity evaluation of bis-histamine Schiff bases and bis-spinaceamine substituted derivatives. Pharm. Chem. J..

[81] Obaid R.J., Mughal E.U., Naeem N., Al-Rooqi M.M., Sadiq A., Jassas R.S., Moussa Z., Ahmed S.A. (2022). Pharmacological significance of nitrogen-containing five and six-membered heterocyclic scaffolds as potent cholinesterase inhibitors for drug discovery. Process Biochem..

[82] Fernandez-Bolanos J.G., Lopez O. (2022). Butyrylcholinesterase inhibitors as potential anti-Alzheimer’s agents: An updated patent review (2018-present). Expert Opin. Ther. Pat..

[83] Begines P., Fernandez-Bolanos J.G., Lopez O. (2026). An updated patent review of acetylcholinesterase inhibitors for the treatment of Alzheimer’s disease (2021–present). Expert Opin. Ther. Pat..

[84] Yıldırım E.R., Güzeldemirci N.U. (2023). Recent advances of cholinesterase inhibitors playing a critical role in the treatment of Alzheimer’s disease (2020–2022). J. Adv. Res. Health Sci..

[85] Lolak N., Tuneğ M., Doğan A., Boğa M., Akocak S. (2020). Synthesis and biological evaluation of 1,3,5-triazine-substituted ureido benzenesulfonamides as antioxidant, acetylcholinesterase and butyrylcholinesterase inhibitors. Bioorg. Med. Chem. Rep..

[86] Kumar S., Paliwal D., Sahu R., Kaushik N. (2025). Recent advancements in the synthesis of pyrazole derivative for the treatment of Alzheimer’s disease. Curr. Org. Chem..

[87] Turkan F., Cetin A., Taslimi P., Gulçin I. (2018). Some pyrazoles derivatives: Potent carbonic anhydrase, α-glycosidase, and cholinesterase enzymes inhibitors. Arch. Pharm..

[88] Shaikh S., Dhavan P., Pavale G., Ramana M.M.V., Jadhav B.L. (2020). Design, synthesis and evaluation of pyrazole bearing α-aminophosphonate derivatives as potential acetylcholinesterase inhibitors against Alzheimer’s disease. Bioorg. Chem..

[89] Biçer A. (2025). Synthesis, AChE and BuChE inhibitory activity, and computational evaluation of pyrazole-based acrylonitrile derivatives. ChemistrySelect.

[90] Zia M., Hameed S., Nadeem H., Kharl A.A., Dege N., Paracha R.Z., Arshad I., Naseer M.M. (2022). Synthesis, structure and acetylcholinesterase inhibition activity of new diarylpyrazoles. Bioorg. Chem..

[91] Li H.-H., Wu C., Zhang S.-L., Yang J.-G., Qin H.-L., Tang W. (2022). Fluorosulfate-containing pyrazole heterocycles as selective BuChE inhibitors: Structure-activity relationship and biological evaluation for the treatment of Alzheimer’s disease. J. Enzym. Inhib. Med. Chem..

[92] Akocak S., Lolak N., Duran H.E., Çetinkaya B.D., Hashem H., Brase S., Türkeş C. (2026). Design and synthesis of 4-arylazo pyrazole carboxamides as dual AChE/BChE inhibitors: Kinetic and in silico evaluation. Pharmaceuticals.

[93] Elmusa M., Elmusa S., Mert S., Kasımoğulları R., Türkan F., Atalar M.N., Bursal E. (2023). One-pot three-component synthesis of novel pyrazolo-acridine derivatives and assessment of their acetylcholinesterase inhibitory properties: An in vitro and in silico study. J. Mol. Struct..

[94] Benazzouz-Touami A., Chouh A., Halit S., Terrachet-Bouaziz S., Makhloufi-Chebli M., Ighil-Ahriz K., Silva A.M.S. (2022). New coumarin-pyrazole hybrids: Synthesis, docking studies and biological evaluation as potential cholinesterase inhibitors. J. Mol. Struct..

[95] Ahmed A.A.M., Mekky A.E.M., Sanad S.M.H. (2025). [3 + 2] Cycloaddition synthesis of new(chromene-1,3,4-oxadiazole) hybrids linked to pyrazole units as potential acetylcholinesterase inhibitors. ChemistrySelect.

[96] Gerni S., Ozturk C., Almaz Z., Bayrak C., Tan A. (2023). Celecoxib derivatives containing pyrazole linked-sulfonamide moiety: Carbonic anhydrase I–II and acetylcholinesterase inhibition profiles, molecular docking studies. ChemistrySelect.

[97] Faudzi S.M.M., Leong S.W., Auwal F.A., Abas F., Wai L.K., Ahmad S., Tham C.L., Shaari K., Lajis N.H., Yamin B.M. (2021). In silico studies, nitric oxide, and cholinesterases inhibition activities of pyrazole and pyrazoline analogs of diarylpentanoids. Arch. Pharm..

[98] Tarikoğulları A.H., Çizmecioğlu M., Saylam M., Parlar S., Alptüzün V., Soyer Z. (2015). Synthesis and cholinesterase inhibitory activity of some phenylacetamide derivatives bearing 1H-pyrazole and 1H-1,2,4-triazole. Marmara Pharm. J..

[99] Kumar A., Ekta N., Kumar V. (2026). Recent advances in carbonyl infused bis-pyrazoles: Relevance, synthetic developments and biological significance. Mol. Divers..

[100] Kumar A., Jhilta P., Brahma M., Kumar M., Kumari P., Singh J., Kumar D., Maruthi M., Ansari A., Singh S. (2026). Design, synthesis, and characterization of new bis-Pyrazoles as potential bioactive agents: Integrated In-vitro and In-silico studies. J. Mol. Struct..

[101] Danne A.B., Dephande M.V., Sangshetti J.N., Khedhar V.M., Shingate B.B. (2021). New 1,2,3-triazole-appended bis-pyrazoles: Synthesis, bioevaluation, and molecular docking. ACS Omega.

[102] Sadeghpour M., Olyaei A. (2021). Recent advances in the synthesis of bis(pyrazolyl)methanes and their applications. Res. Chem. Intermed..

[103] Boraei A.T.A., Soliman S.M., Haukka M., Barakat A., Sarhan A.A.M. (2024). Efficient synthesis and comprehensive characterization of *bis*-pyrazole derivatives: Including X-ray crystallography and Hirshfeld surface analysis. J. Heterocycl. Chem..

[104] Mekky A.E.M., Sanad S.M.H. (2023). [3+2] Cycloaddition synthesis of new piperazine-linked bis(chromene) hybrids possessing pyrazole units as potential acetylcholinesterase inhibitors. Chem. Biodiver..

[105] Mekky A.E.M., Sanad S.M.H. (2023). [3+2] cycloaddition synthesis of new (nicotinonitrilechromene)-based bis(pyrazole) hybrids as potential acetylcholinesterase inhibitors. J. Heterocycl. Chem..

[106] Naglah A.M., Almehizia A.A., Al-Wasidi A.S., Alharbi A.S., Alqarni M.H., Hassan A.S., Aboulthana W.M. (2024). Exploring the potential biological activities of pyrazole-based Schiff bases as anti-diabetic, anti-Alzheimer’s, anti-inflammatory, and cytotoxic agents: In vitro studies with computational predictions. Pharmaceuticals.

[107] Zhang Z., Min J., Chen M., Jiang X., Xu Y., Qin H., Tang W. (2020). The structure-based optimization of δ-sultone-fused pyrazoles as selective BuChE inhibitors. Eur. J. Med. Chem..

[108] Sicak Y., Oruç-Emre E.E., Öztürk M., Karaküçük-İyidoğan A., Nadeem S. (2020). Synthesis, characterization, and antioxidant and anticholinesterase activities of pyrazolo derivatives. J. Heterocycl. Chem..

[109] Gambardella M.D., Wang Y., Pang J. (2025). The Selectivity of butyrylcholinesterase inhibitors revisited. Molecules.

[110] Hanumanthappa D., Kumar B.S., Shajan S.R.O., Sadashivappa N.M., Walikar S.K., Dinesh B.G.H., Ganjipete S., Kunjiappan S., Theivendren P., Chidamabaram K. (2025). Computational insights and experimental breakthroughs in identifying next-generation acetylcholinesterase inhibitors. Sci. Rep..

[111] Islam M.S., Al-Majid A.M., Azam M., Verma V.P., Barakat A., Haukka M., Elgazar A.A., Mira A., Badria F.A. (2021). Construction of spirooxindole analogues engrafted with indole and pyrazole scaffolds as acetylcholinesterase inhibitors. ACS Omega.

[112] Gentile F., Agrawal V., Hsing M., Ton A.T., Ban F., Norinder U., Gleave M.E., Cherkasov A. (2020). Deep Docking: A deep learning platform for augmentation of structure based drug discovery. ACS Cent. Sci..

[113] Herrera-Acevedo C., Perdomo-Madrigal C., Herrera-Acevedo K., Coy-Barrera E., Scotti L., Scotti M.T. (2021). Machine learning models to select potential inhibitors of acetylcholinesterase activity from SistematX: A natural products database. Mol. Divers..

[114] Xiao W., Chen L.Z., Chang J., Xiao Y.W. (2025). Discovery of novel anti-acetylcholinesterase peptides using a machine learning and molecular docking approach. Drug Des. Devel. Ther..

[115] Narayanan S.E., Narayanan H., Mukundan M., Balan S., Vishnupriya C.P., Gopinathan A., Rajamma R.G., Marathakam A. (2021). Design, synthesis and biological evaluation of substituted pyrazoles endowed with brominated 4-methyl 7-hydroxy coumarin as new scaffolds against Alzheimer’s disease. Future J. Pharm. Sci..

[116] Gabr M., Murugan N.A. (2020). Discovery of biphenyl pyrazole scaffold for neurodegenerative diseases: A novel class of acetylcholinesterase-centered multitargeted ligands. Bioorg. Med. Chem. Lett..

[117] Bajad N.G., Jangra J., Kumar A., Krishnamurthy S., Singh S.K. (2025). Discovery of pyrazoline analogs as multi-targeting cholinesterase, β-secretase and Aβ aggregation inhibitors through lead optimization strategy. Int. J. Biol. Macromol..

[118] Waly O.M., El-Sayed S.M., Ghaly M.A., El-Subbagh H.I. (2023). Multi-targeted anti-Alzheimer’s agents: Synthesis, biological evaluation, and molecular modeling study of some pyrazolopyridine hybrids. Eur. J. Med. Chem..

[119] Pravin N., Jozwiak K. (2022). Effects of linkers and substitutions on multitarget directed ligands for Alzheimer’s diseases: Emerging paradigms and strategies. Int. J. Mol. Sci..

[120] Abduljawad A.A., Alkinani K.B., Zaakan A., AlGhamdi A.S., Hamdoon A.A.E., Alshanbari B.H., Alshehri A.A., Alluhaybi B.B., Alqashi S.O.I., Abduljawad R.A. (2025). Targeting amyloid-β proteins as potential Alzheimer’s disease therapeutics: Anti-amyloid drug discovery, emerging therapeutics, clinical trials and implications for public health. Pharmaceuticals.

